# Supplementary material for: The GI Simulated Clinic: A Clinical Reasoning Exercise Supporting Medical Students' Basic and Clinical Science Integration
Source: MedEdPORTAL. 2020 Aug 5;16:10926. doi: 10.15766/mep_2374-8265.10926 (PMC7412764; doi:10.15766/mep_2374-8265.10926)
Supplement: Supplementary file 1 — SP Cases.docxPE Cards.docxLogistics.docxDoor Charts.docxWorksheets.docxDebrief.docxLearner Evaluation.docx [file mep_2374-8265.10926-s001.zip › _Educational_Summary_Report_10926.pdf]

MedEdPORTAL® The AAMC Journal of Teaching and Learning Resources

Original Publication Open Access

The GI Simulated Clinic: A Clinical Reasoning Exercise Supporting Medical Students' Basic and Clinical Science Integration

Donna M. Williams, MD*, Joel T. Bruggen, MD, David E. Manthey, MD, Sharon S. Korczyk, MBA, Jennifer M. Jackson, MD

*Corresponding author: dowillia@wakehealth.edu

Abstract

Introduction: Cognitive integration is required to perform clinical decision-making tasks, even in the preclinical curriculum of medical school. Simulation supports students' cognitive integration by providing practical application of basic science knowledge in a relevant clinical context. To address the need for integrative activities in our curriculum, we implemented a simulated clinic exercise with cases representing gastrointestinal diseases for first-year medical students. **Methods:** Basic science and clinical skills course directors collaborated to design this simulated clinic event, during which student small groups rotated through a series of standardized patient encounters. During each encounter, one student performed the history and physical exam, following which the small group collaboratively developed a prioritized differential diagnosis. Afterwards, the gastroenterology course director debriefed students to highlight key learning points. We collected learner evaluation data following the event. **Results:** Two hundred eighty first-year medical students participated in the simulated clinic in 2018 and 2019. Students rated these events as effective for learning about clinical features of the diseases presented and for reinforcing skills learned in the clinical skills course. Students agreed that the small-group format, pace, and duration were appropriate and that the problem-solving aspect was intellectually stimulating. The most effective aspects were opportunities to solidify illness scripts, apply knowledge to solve a problem, and encounter diseases in a realistic clinical context. **Discussion:** This simulated clinic model effectively supported preclinical students' basic and clinical science integration to complete diagnostic reasoning tasks for gastrointestinal gastrointestinal conditions and was evaluated favorably by learners.

Keywords
Simulation, Physical Exam, Abdominal Pain, Standardized Patient, Hypothesis-Driven Data Gathering, Differential Diagnosis, Gastroenterology, Primary Care, Case-Based Learning

Educational Objectives

By the end of this activity, learners will be able to:

1. Use hypothesis-driven data gathering to identify key or distinguishing features of a patient's clinical presentation in a time-limited encounter.
2. Interpret physical exam findings to further characterize a patient's problem representation.
3. Compare and contrast a patient's problem representation with their own illness scripts to formulate an appropriate, prioritized differential diagnosis.
4. Suggest appropriate diagnostic testing based on their differential diagnosis.

Citation:
Williams DM, Bruggen JT, Manthey DE, Korczyk SS, Jackson JM. The GI simulated clinic: a clinical reasoning exercise supporting medical students' basic and clinical science integration. *MedEdPORTAL*. 2020;16:10926. https://doi.org/10.15766/mep_2374-8265.10926

Introduction

Physicians must cognitively integrate and apply knowledge from multiple disciplines in order to make appropriate diagnostic and patient management decisions. Cognitive integration is required as early as the preclinical curriculum of medical school, including on high-stakes assessments such as the United States Medical Licensing Examination (USMLE) Step 1 board exam, during which vignette-based questions require students to quickly assimilate and analyze basic science and clinical information in order to answer questions. During their clerkship training, medical students are expected to perform a variety of tasks requiring effective cognitive integration, including formulation of differential diagnoses, recognition of emergent medical conditions, and development of patient-centered diagnostic and management plans. In addition, per the Liaison Committee on

Copyright © 2020 Williams et al. This is an open-access publication distributed under the terms of the Creative Commons Attribution license.

Medical Education, it is required that a medical school curriculum "provides opportunities for medical students to acquire skills of critical judgment based on evidence and experience, and develops medical students' ability to use those principles and skills effectively in solving problems of health and disease."[1]

Thoughtful instructional design is needed to ensure opportunities for students to engage in cognitive integration of basic and clinical sciences.[2,3] Simulation has frequently been employed in preclinical medical school curricula, though most schools have used it to teach clinical skills, and few have used it for basic science integration.[4-6] Simulation supports cognitive integration by providing an explicit, practical application of basic science knowledge in a relevant clinical context,[7] offering learners opportunities to develop their causal knowledge—a strategy utilized by expert clinicians in which basic science knowledge is used to explain clinical presentations of disease.[8-11] Simulation also leads to high levels of learner engagement—a strong predictor of learning outcomes.[12,13] As an instructional method, simulation is supported by Kolb's experiential learning theory, which asserts that learning is grounded in experience, active involvement is key to learning, and learning involves transactions between the learner and the environment.[14]

To address the desire for integrative activities in our preclinical curriculum, we developed a simulated clinic learning activity to provide first-year medical students with opportunities to practice hypothesis-driven data-gathering skills and reinforce illness scripts for diseases encountered in the gastroenterology (GI) course. Studies on diagnostic accuracy of physicians indicate that the ability to recognize important diagnostic cues from the patient history correlates with the ability to correctly diagnose the etiology of patients' presenting complaints.[15,16] Therefore, the ability to collect a diagnostically relevant and accurate history is a crucial skill for medical trainees.[17] In terms of the physical exam (PE), Yudkowsky and colleagues showed that shifting from a complete, head-to-toe PE to a hypothesis-driven PE is a reliable method to teach and assess PE skills as well as to incorporate clinical reasoning into the patient assessment.[18] Staitieh, Saghafi, Kempker, and Schulman also found that teaching hypothesis-driven PEs to learners improves their ability to interpret PE findings.[19]

The simulated GI clinic activity consisted of four standardized patient (SP) cases: appendicitis presenting as acute abdominal pain, cholecystitis presenting as acute abdominal pain, ulcerative colitis presenting as hematochezia, and pancreatic cancer presenting as poor appetite and weight loss. Due to the limited knowledge and experience of the learner group, we chose clinical presentations of leading and well-known diagnoses to prevent an overwhelming cascade of differentials. This allowed students to start practicing skills, such as illness script identification, analytical reasoning, compare-and-contrast strategies, and hypothesis testing, in recognizable clinical scenarios. Case-based learning resources representing these GI conditions—and others presenting with similar clinical features (e.g., abdominal pain)—have been published previously. The following is a summary of those resources, with a description of the existing gaps in instructional delivery for clinical reasoning practice among them.

Several such resources have been developed involving a number of active learning methods that do not feature interaction with SPs. Denham and Chuang's small-group, case-based activity on abdominal pain for second-year medical students has been designed to promote diagnostic reasoning skills.[20] Because this activity is presented as a written case, providing history and PE data to learners rather than allowing them to select and elicit the data from a patient, learners cannot practice hypothesis-driven data gathering during the activity. Students can practice developing a differential diagnosis, but some guidance is provided in the worksheet on organ systems to consider for the case, rather than allowing students to independently decide on disease categories for their differential diagnosis. Woosley, Brady, and Boland developed an online, self-directed acute abdominal pain case representing appendicitis, designed to promote clinical reasoning among individual students working independently.[21] Because it provides history and PE data to learners rather than allowing them to select and elicit the data from a patient, learners cannot use this tool to practice hypothesis-driven data gathering. Fishman published a case of a young man with inflammatory bowel disease, but the scenario has been written for experienced clinicians rather than preclinical students, focuses primarily on clinician-patient communication skills, and is implemented as a written case for small-group discussion rather than as an SP exercise.[22] Other authors have published team-based learning exercises on GI conditions for preclinical medical students, some of which overlap with the four GI conditions included in this resource.[23,24]

Several authors have published SP-based activities related to GI pathology as well. DeSipio, Gaughan, Perlis, and Phadtare describe a learning activity involving SPs to teach preclinical medical students during the GI block, though the SPs are used to demonstrate aspects of GI tract anatomy through an endoscopy demonstration rather than for students' clinical skills practice.[25] Mulligan and colleagues' SP-based learning activity involves evaluation of geriatric patients with weight loss; however, it is

Copyright © 2020 Williams et al. This is an open-access publication distributed under the terms of the Creative Commons Attribution license.

designed as an interprofessional team activity with team-oriented learning goals and practice using a specific screening tool rather than diagnostic reasoning.[26] Akins[27] and Krugler[28] have each published SP cases of acute abdominal pain representing acute cholecystitis intended for use with medical students. In both publications, an SP script and student performance evaluation form are included. Akins' resource also includes a postencounter worksheet designed to serve as preparation for an oral presentation, but it does not include practice comparing and contrasting the conditions on the differential diagnosis with the patient's clinical presentation. Falcone and Ogilvie have published a series of three SP cases involving abdominal pain, intended for students' formative practice in preparation for the USMLE Step 2 CS exam.[29] Although their SP exercises were developed similarly to those we describe in this resource, the GI conditions represented are different (cholangitis, diverticulitis, and perforated ulcer). Terregino, Corbett, Crocco, Carman, and Saks developed a set of SP cases representing pathology across multiple organ systems, a subset of which represents five GI conditions (inflammatory bowel disease, acute appendicitis, obstructive jaundice due to pancreatic cancer, gastroesophageal reflux disease, and colon cancer). In their exercise, 10-student teams rotate together to each SP encounter, two students collect history and PE data in each encounter, and only history is collected directly from SPs, as PE data are instead elicited from faculty facilitators. With this model, individual student participation in hypothesis-driven data collection is limited, and PE skills are not practiced. Differential diagnosis formation skills can be practiced, but a tool facilitating compare/contrast between the differential diagnosis list and each patient case is not provideds.[30]

In the following resource, we developed a collaborative learning exercise in which individual students have opportunities to directly practice hypothesis-driven history taking and PE skills with SPs, while student small groups can practice identifying the illness scripts represented in each encounter by using a structured compare-and-contrast process, outlined in a diagnostic reasoning worksheet. Through this diagnostic reasoning exercise, students practice integrating new knowledge from their GI course with data collection skills from their clinical skills course. Students receive feedback on their diagnostic reasoning performance through a structured debriefing led by the GI course director.

Methods

Educational Context

This learning activity was designed to provide first-year medical students in the GI course with an opportunity to integrate their newly acquired knowledge of GI diseases with their data-gathering skills in order to practice their diagnostic reasoning skills. The GI course director (Joel T. Bruggen) and clinical skills course directors (Donna M. Williams, Jennifer M. Jackson) collaborated to design this simulated clinic event, including the learning objectives, the corresponding learner tasks, the SP cases, and the logistical plan. These individuals collaborated with the director of the medical decision-making thread (David E. Manthey) to design a worksheet to facilitate students' practice of differential diagnosis prioritization through an explicit comparing and contrasting of the patient's clinical presentation with each of the conditions on the differential diagnosis.

Learner Prerequisites

Prerequisites for learners to participate in this activity included some exposure to the GI pathology relevant to the chief complaints presented in the cases, basic history taking skills, and abdominal examination skills, including the basic screening exam of the abdomen—inspection, auscultation, general palpation, liver percussion, and liver and spleen palpation—as well as additional abdominal maneuvers to look for specific abdominal pathology (e.g., maneuvers to look for evidence of appendicitis).

Event Preparation and Space Setup

SP recruitment and training: SPs were recruited by the SP coordinator based on the demographic features of each case and in sufficient numbers for the planned number of exam rooms for this event. SPs were sent a copy of their assigned case in advance of the event. An SP training session was conducted by the GI course director, who reviewed all case details with the SPs and fielded questions (Appendix A: SP Cases). For cases involving one or more abnormal PE findings, the SPs for those cases were given PE cards with corresponding instructions regarding when and if to provide these findings to students (Appendix B: PE Cards). The SPs were instructed to give the card to the student only if the PE maneuver reflected on the card was performed correctly. Of note, some of the SP case scenarios only involved PE findings that were acted out by the SPs (e.g., abdominal tenderness) rather than having any PE cards. For the cases requiring the SPs to act out tenderness with palpation, the specific locations and degree of tenderness were reviewed in detail during the training session.

Logistics:

- Space planning: This learning activity took place in our school's simulation center, which included a group of simulated ambulatory and inpatient exam rooms. SPs were assigned to specific rooms according to our logistical plan.

Copyright © 2020 Williams et al. This is an open-access publication distributed under the terms of the Creative Commons Attribution license.

Since we used four cases for this activity, we assigned five SPs to each case and used 20 of our simulation center's exam rooms. This enabled us to host a large group of students simultaneously (note that based on available facilities, this activity could be adjusted for different numbers of learners). Cases were arranged in the exam rooms so that students could rotate from one room to the adjacent room at the end of each encounter, thus minimizing transition times.

- Student scheduling and group assignments: Students were divided into two large subgroups for this event; each subgroup participated in this activity during an assigned 2-hour time frame. Students in each subgroup were divided into assigned small groups of three to four students each.

Of note, the event duration can be adjusted according to the learner group size, one's space needs and availability, and desired number of cases. See Appendix C: Logistics for logistics details and event design options.

Exam room preparation—door charts: A simulated patient chart for each case was placed outside of each exam room for student review prior to entering the room (Appendix D: Door Charts). This document included the patient's name, age, chief complaint, and vital signs for the case.

Learner orientation: A few days prior to the activity, the clinical skills and GI course Directors sent students an informational email with instructions on expected dress (professional), a brief overview of the logistics of the event, and students' instructions for the activity. Students were asked to report to the simulation center 10-15 minutes prior to their assigned simulated clinic time for attendance sign-in, to receive their assigned exam room rotation schedule, and to obtain their small group's worksheet packet (Appendix E: Worksheets).

Event Implementation

Student check-in: Upon check-in prior to the activity start, each small group of students was given an assigned schedule for the group's rotation among rooms (Appendix C: Logistics) as well as a worksheet packet (Appendix E: Worksheets).

Brief learner orientation: Immediately prior to the activity, one of the clinical skills course directors briefly reviewed with students their instructions for the activity (see Appendix C: Logistics for details).

SP encounter series: Student small groups then began the SP encounters. Twenty minutes were allocated for data gathering (history taking and PE) for each case, and an additional 10 minutes were allocated for small-group discussion and worksheet completion immediately following each encounter. Students were instructed to assign one student to lead the history and PE portion of each encounter, so that each student had an opportunity to lead the interview and PE of at least one SP encounter. Students were then instructed to work as a team to formulate their differential diagnosis, suggested diagnostic workup, and rationale for both on the worksheet corresponding to each case. The worksheets were intended to guide the students' approach to each patient by comparing and contrasting the diseases on their differential diagnosis to the patient's case.

Rotation conclusion: At the end of the small-group rotation series, students submitted their worksheet packets to staff, who provided these documents to the GI course director. As this event was formative in nature, not summative, scoring of individual student groups' performance on the worksheets was not required, and large-group feedback was provided within the debrief (see below).

Simulation debrief: The day following the simulated GI clinic, the GI course director met with students as a large group to review the case series. The debrief was scheduled on the day after the event so that it was as close to the event as possible, for optimal student recall of case details. Approximately 1 hour was allocated for this activity. Using commercially available polling software, the GI course director went through questions about each case (see Appendix F: Debrief for specific questions used). Each question was used as a springboard for further interactive discussion in the large-group setting, especially when the poll results indicated incomplete understanding. The answers to every question about each case and the reasons behind them were reviewed when identified as necessary. In this way, the key and distinguishing features of each case scenario were emphasized for the group at large. Ideally, the debrief facilitator would have reviewed all student worksheets prior to the debrief session; however, due to time constraints, this was not possible.

Program Evaluation

We collected learner evaluation data from participating students through an anonymous, online, voluntary survey following the event (Appendix G: Learner Evaluation).

Results

One hundred thirty-eight first-year medical students participated in the event in April 2018, and 141 first-year students participated in the event in April 2019.

After the first iteration in 2018, students felt that the simulated GI clinic activity was a helpful exercise but that the logistics needed

Copyright © 2020 Williams et al. This is an open-access publication distributed under the terms of the Creative Commons Attribution license.

some adjustment. Students' narrative feedback noted the most effective aspects of this activity as being the opportunities it provided to solidify illness scripts for the diseases represented in the SP cases, to apply knowledge to solve a problem, and to encounter diseases in a realistic clinical context. Suggestions for improvement included increasing the time between cases to discuss patients with peers, guidance on structuring students' time with the SPs during the encounters, and improved debriefing. We used student feedback on the first simulated clinics to improve subsequent events.

The postevent learner evaluation results for the first (April 2018) and second (April 2019) implementations of this event are presented in the Table.

Discussion

Simulation supports integration by providing learners with opportunities for explicit, practical application of basic science knowledge in a relevant clinical context.[7] Simulation also leads to high levels of learner engagement where "learning occurs in a highly activated state."[7] The GI simulated clinic was feasible and straightforward to plan, was time efficient, required only one on-site faculty, and was low cost. Students found this instructional format to be effective and engaging. This model is highly versatile and can be tailored to achieve various educational goals. The core knowledge and clinical skills addressed in the GI simulated clinic are relevant to medical student trainees at large, and therefore, the relevance and applicability of this activity are generalizable to educators and students at other institutions.

Lessons Learned

The logistical plan for this type of learning activity needs to be carefully selected, based on the overall goals of the simulated clinic exercise. This includes determining the timing of the event in students' academic calendar, the number of SP cases, the duration of each encounter, the amount of time allotted for small-group discussion about the differentials and diagnostic plan, and the debriefing format and timing. From students' feedback following the first implementation of this activity in 2018, we learned a lot that we later applied to making revisions to its design in 2019.

The first year we implemented this activity, it was timed in the middle of the students' GI basic science course block, at which point they had only learned about a portion of the diseases in this organ system. However, following that event, students requested that it be timed later in the GI block so that they could learn about more diseases that they might consider in their differential diagnoses for the cases in this activity. So, we scheduled the event accordingly for the second iteration the following year.

In the first iteration of this activity, we used five cases, a number that most students felt was appropriate given the time allotted. However, there was some variation in student preferences, with some requesting more cases (to cover more disease conditions) and some requesting fewer (to provide more time to work on each case). There are pros and cons to both approaches, so this decision can be determined based on the individual educator's primary learning objectives for the learners and based on the availability and number of the spaces used for this event.

Table. Learner Evaluation Results of Simulated Clinic, 2018 (*N* = 25, 18% Response Rate) and 2019 (*N* = 55, 39% Response Rate)

	Yes (%)		No (%)		Extremely (%)		Quite (%)		Moderately (%)	
**Question**	**2018**	**2019**	**2018**	**2019**	**2018**	**2019**	**2018**	**2019**	**2018**	**2019**
How relevant was the content of this learning activity to your role as a future physician?					68	73	28	25	0	2
Was the small-group format of this learning activity appropriate for the learning content presented?	100	100	0	0						
Were the pace and duration of this learning activity appropriate for the learning content presented?	92	95	8	5						
Was the problem-solving aspect of this activity intellectually stimulating?	96	98	4	2						
Do you prefer classes that include this type of small-group problem-solving activity rather than classes that do not include this type of activity?	92	98	8	2						
Effectiveness of the SP encounters for learning about the clinical features of the diseases presented in this learning activity?					60	60	20	33	16	5
Effectiveness of the SP encounters for practicing your diagnostic reasoning skills related to the diseases presented in this learning activity?					64	76	28	22	4	2
Effectiveness of the SP interviews for reinforcing history taking skills you have learned in the Clinical Skills 1 course?					60	75	24	16	12	7
Effectiveness of the SP encounters for reinforcing PE skills you have learned in the Clinical Skills 1 course?					60	69	24	22	4	9

Abbreviations: PE, physical exam; SP, standardized patient.

Copyright © 2020 Williams et al. This is an open-access publication distributed under the terms of the Creative Commons Attribution license.

Based on student feedback in which students contrasted their experience in this event to another simulated clinic activity earlier in the year whose logistics were slightly different, we learned that the size of learner small groups for this activity was important: Students preferred the smaller group size used for this activity, with an optimal group size being three to four students. This size allowed each student an opportunity to take turns leading at least one of the SP encounters, which better engaged all students in each group over the course of the activity.

The time allotted for students to discuss each case with their small group was also an important design element, per students' feedback. They preferred having this time immediately following each encounter (vs. at the very end of the whole exercise, which had been the case in a previous simulated clinic event), and they requested more than just the 5 minutes allotted in our first iteration of this event, to allow their group enough time for a meaningful discussion of each case. Therefore, we increased the small-group discussion time following each case to 10 minutes in our second iteration of the exercise.

The format and timing of the debriefing are also important instructional design considerations for this type of learning activity. Providing a timely debriefing to students—as soon after the event as possible, while the cases are fresh in their minds—is ideal. In addition, students preferred having a structured debriefing on the case series to affirm or redirect their diagnostic conclusions rather than simply an answer sheet with the correct disease conditions listed, since identifying the key clinical features of each case was just as important to them as identifying the correct answer.

Limitations

This resource does require access to sufficient exam room space, with facilities to permit both history taking and PE practice, as well as some funding to hire SPs. Assessing students' worksheets requires some time and is helpful to do prior to delivering the debriefing session to the students, but may or may not be possible if the debriefing is scheduled immediately following the SP case series. Due to time constraints, individual feedback on worksheets was not provided. Aside from evaluating students' evaluation of the event, other methods to evaluate the objectives, such as pre- and posttesting to assess change in knowledge or skill, were not performed.

Future Directions

Future directions for this work include utilizing the simulated clinic format to create similar learning events in additional basic science courses during the preclinical curriculum as ongoing diagnostic reasoning practice for these early learners, as well as implementing these cases within an interprofessional education setting with other learner groups, such as physician assistant students, more-advanced medical students, or residents. Future iterations could include electronic versions of the worksheets to allow for easier review by course faculty. Additional work is needed to determine if this instructional approach results in measurable learning outcomes indicating improvement in these early learners' diagnostic reasoning development.

Appendices

A. SP Cases.docx
B. PE Cards.docx
C. Logistics.docx
D. Door Charts.docx
E. Worksheets.docx
F. Debrief.docx
G. Learner Evaluation.docx

All appendices are peer reviewed as integral parts of the Original Publication.

Donna M. Williams, MD: Associate Professor, Department of Internal Medicine, Wake Forest School of Medicine

Joel T. Bruggen, MD: Professor, Section of Gastroenterology, Department of Internal Medicine, Wake Forest School of Medicine

David E. Manthey, MD: Professor, Department of Emergency Medicine, Wake Forest School of Medicine

Sharon S. Korczyk, MBA: Curriculum Coordinator, Academic Affairs, Wake Forest School of Medicine

Jennifer M. Jackson, MD: Associate Professor, Department of Pediatrics, Wake Forest School of Medicine

Disclosures
None to report.

Funding/Support
None to report.

Prior Presentations
Williams D, Peters T, Jackson J. Orchestrating the "aha" moment: a simulated clinic for basic and clinical science integration. Poster presented at: Directors of Clinical Skills Precourse, Learn Serve Lead: The AAMC Annual Meeting; November 2, 2018; Austin, TX.

Ethical Approval
Reported as not applicable.

Copyright © 2020 Williams et al. This is an open-access publication distributed under the terms of the Creative Commons Attribution license.

References

1. Liaison Committee on Medical Education. *Functions and Structure of a Medical School: Standards for Accreditation of Medical Education Programs Leading to the MD Degree*. Liaison Committee on Medical Education; 2018.
2. Kulasegaram KM, Martimianakis MA, Mylopoulos M, Whitehead CR, Woods NN. Cognition before curriculum: rethinking the integration of basic science and clinical learning. *Acad Med*. 2013;88(10):1578-1585. https://doi.org/10.1097/ACM.0b013e3182a45def
3. Goldman E, Schroth WS. Deconstructing integration: a framework for the rational application of integration as a guiding curricular strategy. *Acad Med*. 2012;87(6):729-734. https://doi.org/10.1097/ACM.0b013e318253cad4
4. Passiment M, Sacks H, Huang G. Medical simulation in medical education: results of an AAMC survey. Association of American Medical Colleges. Accessed May 6, 2018. https://www.aamc.org/download/259760/data
5. Ginzburg SB, Brenner J, Cassara M, Kwiatkowski T, Willey JM. Contextualizing the relevance of basic sciences: small-group simulation with debrief for first- and second-year medical students in an integrated curriculum. *Adv Med Educ Pract*. 2017;8:79-84. https://doi.org/10.2147/AMEP.S124851
6. Takkunen M, Turpeinen H, Viisanen H, Wigren HK, Aarnio M, Pitkäniemi J. Introduction of real patients into problem-based learning in preclinical first-year anatomy curriculum. *Med Teach*. 2011;33(10):854-856. https://doi.org/10.3109/0142159X.2011.576718
7. Eason MP. The use of simulation in teaching the basic sciences. *Curr Opin Anaesthesiol*. 2013;26(6):721-725. https://doi.org/10.1097/ACO.0000000000000008
8. Gordon JA, Hayden EM, Ahmed RA, Pawlowski JB, Khoury KN, Oriol NE. Early bedside care during preclinical medical education: can technology-enhanced patient simulation advance the Flexnerian ideal? *Acad Med*. 2010;85(2):370-377. https://doi.org/10.1097/ACM.0b013e3181c88d74
9. Schmidt HG, Rikers RMJP. How expertise develops in medicine: knowledge encapsulation and illness script formation. *Med Educ*. 2007;41(12):1133-1139. https://doi.org/10.1111/j.1365-2923.2007.02915.x
10. Krynski TR, Tenenbaum JB. The role of causality in judgment under uncertainty. *J Exp Psychol Gen*. 2007;136(3):430-450. https://doi.org/10.1037/0096-3445.136.3.430
11. Holyoak KJ, Cheng PW. Causal learning and inference as a rational process: the new synthesis. *Annu Rev Psychol*. 2011;62: 135-163. https://doi.org/10.1146/annurev.psych.121208.131634
12. Appleton JJ, Christenson SL, Furlong MJ. Student engagement with school: critical conceptual and methodological issues of the construct. *Psychol Sch*. 2008;45(5):369-386. https://doi.org/10.1002/pits.20303
13. Wang MT, Degol J. Staying engaged: knowledge and research needs in student engagement. *Child Dev Perspect*. 2014;8(3):137-143. https://doi.org/10.1111/cdep.12073
14. Kolb DA. *Experiential Learning: Experience as the Source of Learning and Development*. Prentice Hall; 1984.
15. Kostopoulou O, Oudhoff J, Nath R, et al. Predictors of diagnostic accuracy and safe management in difficult diagnostic problems in family medicine. *Med Decis Making*. 2008;28(5):668-680. https://doi.org/10.1177/0272989X08319958
16. Hertwig R, Meier N, Nickel C, et al. Correlates of diagnostic accuracy in patients with nonspecific complaints. *Med Decis Making*. 2013;33(4):533-543. https://doi.org/10.1177/0272989X12470975
17. Henderson MC, Tierney LM Jr, Smetana GW. *The Patient History: An Evidence-Based Approach to Differential Diagnosis*. 2nd ed. McGraw-Hill; 2012.
18. Yudkowsky R, Otaki J, Lowenstein T, Riddle J, Nishigori H, Bordage G. A hypothesis-driven physical examination learning and assessment procedure for medical students: initial validity evidence. *Med Educ*. 2009;43(8):729-740. https://doi.org/10.1111/j.1365-2923.2009.03379.x
19. Staitieh BS, Saghafi R, Kempker JA, Schulman DA. Teaching a hypothesis-driven physical diagnosis curriculum to pulmonary fellows improves performance of first-year medical students. *Ann Am Thorac Soc*. 2016;13(4):489-494. https://doi.org/10.1513/AnnalsATS.201505-297OC
20. Denham A, Chuang A. Young woman with abdominal pain: a case-based exercise for medical students. *MedEdPORTAL*. 2012;8:9140. https://doi.org/10.15766/mep_2374-8265.9140
21. Woosley J, Brady C, Boland K. Young man with abdominal pain - an interactive case exercise. *MedEdPORTAL*. 2010;6:7764. https://doi.org/10.15766/mep_2374-8265.7764
22. Fishman L. But Tommy likes it here: moving to adult medicine. *MedEdPORTAL*. 2012;8:9190. https://doi.org/10.15766/mep_2374-8265.9190
23. Bass RZ, Morgan DE, Brooks WS. A case of pancreatic cancer: abdominal anatomy team-based learning module for medical students. *MedEdPORTAL*. 2018;14:10700. https://doi.org/10.15766/mep_2374-8265.10700
24. Bishop S, Kushinka J, Call S. Internal Medicine Clerkship Team-Based Learning Series: abdominal pain module. *MedEdPORTAL*. 2012;8:9163. https://doi.org/10.15766/mep_2374-8265.9163
25. DeSipio J, Gaughan J, Perlis S, Phadtare S. Use of real patients and patient-simulation-based methodologies for teaching gastroenterology to pre-clinical medical students. *Healthcare (Basel)*. 2018;6(2):61. https://doi.org/10.3390/healthcare6020061
26. Mulligan R, Gilmer-Scott M, Kouchel D, et al. Unintentional weight loss in older adults: a geriatric interprofessional simulation case series for health care providers. *MedEdPORTAL*. 2017;13:10631. https://doi.org/10.15766/mep_2374-8265.10631

Copyright © 2020 Williams et al. This is an open-access publication distributed under the terms of the Creative Commons Attribution license.

27. Akins R. Victor Billar, acute abdominal pain. *MedEdPORTAL*. 2012;8:9118. https://doi.org/10.15766/mep_2374-8265.9118

28. Krugler J. "Acute cholecystitis" case for SPs. *MedEdPORTAL*. 2006;2:145. https://doi.org/10.15766/mep_2374-8265.145

29. Falcone J, Ogilvie J. Three adult acute abdominal pain objective structured clinical examination (OSCE) cases for medical student assessment in the surgery clerkship. *MedEdPORTAL*. 2011;7:8139. https://doi.org/10.15766/mep_2374-8265.8139

30. Terregino C, Corbett S, Crocco J, Carman R, Saks N. Cardinal manifestations and presentation of disease—making the rounds on standardized patients with alterations in cardiac, respiratory, gastrointestinal, and genitourinary function. *MedEdPORTAL*. 2008;4:1132. https://doi.org/10.15766/mep_2374-8265.1132

Received: August 12, 2019
Accepted: January 10, 2020
Published: August 5, 2020

Copyright © 2020 Williams et al. This is an open-access publication distributed under the terms of the Creative Commons Attribution license.
