# Supplementary material for: The GI Simulated Clinic: A Clinical Reasoning Exercise Supporting Medical Students' Basic and Clinical Science Integration
Source: MedEdPORTAL. 2020 Aug 5;16:10926. doi: 10.15766/mep_2374-8265.10926 (PMC7412764; doi:10.15766/mep_2374-8265.10926)
Supplement: Supplementary file 1 — SP Cases.docxPE Cards.docxLogistics.docxDoor Charts.docxWorksheets.docxDebrief.docxLearner Evaluation.docx [file mep_2374-8265.10926-s001.zip › A. SP Cases.docx]

Appendix A: Standardized Patient Case Development Tool (Cases 1-4)

*Case 1:* *Appendicitis*

Date: July 29, 2019

Primary Case Author: Donna Williams, MD

Secondary Case Author: Joel Bruggen, MD

Standardized Patient Educator: Donna Williams, MD and Joel Bruggen, MD

Name of Case: Appendicitis

Name of educational and or assessment activity: GI Simulated Clinic

Patient Name: Jack Simmons

Chief Complaint: “my stomach hurts”

Most likely Diagnosis and Differential with rationale from history and/or physical exam: This patient has acute appendicitis. This is the most likely diagnosis based on the patient’s report of worsening abdominal pain that began around the umbilicus and settled into the right lower quadrant with associated anorexia, nausea, and fever. The physical exam findings of tenderness at McBurney’s point as well as the positive Rovsing’s and Psoas Signs are also suggestive of acute appendicitis. Other diagnoses to consider include Crohn’s Disease, peptic ulcer disease, kidney stone, and acute pancreatitis.

Challenge question: None

Domains: Check all that apply

- Professionalism

X Communication and Interpersonal skills

X Medical History

X Physical exam

- Shared Decision Making
- Patient Education

X Clinical Reasoning

- Documentation
- Handoff
- Presentation
- Other:

Type and level of learner: First or second year medical students

Case Objectives:

1. Medical History:
   1. Use hypothesis-driven data gathering to identify key or distinguishing features of a patient’s clinical presentation in a time-limited encounter.
2. Physical Exam:
   1. Interpret physical exam (PE) findings to further characterize a patient’s problem representation.
3. Clinical Reasoning:
   1. Compare and contrast a patient’s problem representation with one’s illness scripts to formulate an appropriate, prioritized differential diagnosis.
   2. Suggest appropriate diagnostic testing based on one’s differential diagnosis.

| SETTING: outpatient, in patient, ED, home, nursing home, rehab, group etc. | Emergency Department |
| --- | --- |
| PATIENT PROFILE: Information about the “patient” that helps select an SP and helps the learner get an understanding of them as a person. SP will know more information about the patient than learner will ever ask but allows SP to portray a fully developed patient personality. If none of the items below are particulars for the case please write “all may be used.” | |
| Age range | 18-22 years |
| Religious/spiritual background | Any |
| Sex (e.g., male, female, intersex, transwoman, transman) | Male |
| Sexual Orientation (e.g., heterosexual, lesbian, gay, bisexual, pansexual, queer, asexual) | Any |
| Gender expression (e.g., man, woman, gender queer) | Male |
| Race/ethnicity: | Any |
| Physical description (e.g., BMI, height range) | Average BMI |
| Physical limitations | None |
| Patient appearance (e.g., disheveled, hospital gown, business casual, casual) | Hospital gown |
| Moulage + location (e.g., none, bruises, scars, body piercing, tattoos) | None |
| Affect (e.g., pleasant, cooperative) | Pleasant, cooperative |
| Family group (e.g., who is family, who they live with) | You live with your parents; you have one older sister who lives in an apartment nearby. |
| Education | Graduated high school, some college completed. You are currently studying computer science. |
| Level of health literacy | Average |
| Employment, if any - present and past, noting any current stresses | You work a part time job at the grocery store while you are going to school. |
| Home/homeless - type of dwelling, number of stories, owned or rented | You live in a single family home with your parents. |
| Financial situation- any current stresses | No current stresses. |
| Insurance Status (e.g., un/under/insured, public/private, HMO/PPO) | You are insured under your parents’ health insurance. |
| Habits (i.e., diet, exercise, caffeine, smoking, alcohol, drugs) | Diet: You eat a typical diet of pizza, sandwiches, hamburgers for lunch and a healthy dinner that your Mom cooks at night  Exercise: You exercise at the gym a couple days per week and like to stay active (running, biking)  Tobacco: none  Alcohol: occasional beer on the weekends at parties (max of 4 drinks per party, usually 2-3)  Drugs: none |
| Activities (i.e., hobbies, sports, clubs, friends) | You enjoy working out at the gym and hanging out with your friends. |
| Typical day - what is the usual daily routine | On a typical day, you got to classes at community college, where you are studying computer science. Some days you also work at your part time job at the grocery store. |

| CASE INFORMATION | |
| --- | --- |
| Chief Concern: What the patient will say when greeted by the student. The patient’s primary reason for seeking medical care often stated in his/own words. | My stomach is really bothering me; it started yesterday and hurts so bad that I thought I should get it checked out. |
| Additional Concerns: Other, if any, concerns the patient has today (i.e., symptoms, requests, expectations, etc.) that will become part of set agenda. | None |
|  | |
| THE PATIENT STORY: The SP will be asked to tell their symptom story and the personal and emotion impact for each of their concerns. You will want to write this is the patient voice. The symptom story should be able to answer this question: “Tell me more about [chief concern/additional concern], starting at the beginning and bringing me up to now.”  The personal context should be able to answer questions concerning the broader personal/psychosocial context of symptoms, especially the patient beliefs/attributions.  The emotional context should be able to ask how are you doing with this, how does this make you feel, how has this affected you emotionally? IMPACT: How has this affected your life? How has this been for your family? | I was feeling fine until yesterday afternoon. I had pizza for lunch with some friends and had some indigestion afterwards. At least, that’s what I thought it was, I’ve never really had indigestion before. Last night, one of people I was with texted me to say that he was throwing up, so I wonder if I caught something from him. I didn’t feel like eating last night, so haven’t really had anything to eat since lunch yesterday. I woke up this morning at 6am and had this bad pain in my stomach around my belly button. |
| HISTORY OF PRESENT ILLNESS: Although some of the HPI will be given in the patient’s symptom story, the learners will expand the story during the direct question section. Below describe the detailed history, usually about the chief concern, which the student must develop in order to make a useful assessment of the problem: | |
|  | |
| Onset (when; gradual or sudden) | Started yesterday after lunch, gradually getting worse |
| Setting (what was going on or where was patient when symptoms first noticed?) | Noticed after eating pizza yesterday at lunch |
| Duration (how long) | About one day |
| Time relationships (frequency, constant or intermittent) | Pain is constant |
| Location | The pain started around my belly button, but now seems like it’s worse on the lower right side of my belly. |
| Radiation | None |
| Quality | It started out dull, but now feels sharp |
| Amount | Severity initially was 3 out of 10, now it’s up to a 6 |
| Aggravated by what | Coughing makes the pain worse. I haven’t tried to eat or drink anything, but I think that would make it worse. |
| Relieved by what | Nothing. I tried taking some Tylenol, but that didn’t help at all. |
| Associated with what | - Nausea: Since I woke up this morning, I’ve felt nauseous, but haven’t vomited yet. The nausea seems to come in waves, and nothing seems to really make it better or worse.  - Fever: When I woke up this morning I felt hot and sweaty. I’ve felt like I have a fever since then, but I don’t have a thermometer so I haven’t taken my temperature. |
| Attitude (what does the patient think is the problem, and how does he/she feel about it) | I wonder if I got some kind of stomach bug from my friend, but the pain is getting worse so I’m not sure |
| Overall course | Consistently worsening since pain began yesterday afternoon |
| REVIEW OF SYSTEMS: Significant positives and negatives | |
| Fever | When I woke up this morning I felt hot and sweaty. I’ve felt like I have a fever since then, but I don’t have a thermometer so I haven’t taken my temperature. |
| Chills/Night Sweats | None |
| Vomiting/Diarrhea/Constipation/Stool Changes | None |
|  |  |
|  | |
| Past medical history | Seasonal allergies, mild intermittent asthma |
| Medication allergies (Name and reaction) | None |
| Environmental allergies (Name and reaction) | Grass – sneezing, itchy eyes, runny nose |
| Illnesses | None |
| Vaccinations | I’ve had all my regular vaccines, as far as I know |
| Surgeries | None |
| Accidents/ injuries/ trauma | None |
| Hospitalization | None |
|  | |
| Inclusive sexual and reproductive history | |
| Sexual practices  Sexual partners  Protection: Use of safer sex practices  Use of birth control if appropriate  Risk of intimate partner violence | Heterosexual  One current sexual partner (one lifetime partner)  Use condoms for protection |
| Ob/GYN HISTORY | Age of onset of menses N/A  Age of menopause  Number of pregnancies  Number of live births  Number of miscarriages  Number of abortions |
| Medications | Prescription/dose/reason  - Albuterol inhaler, 2 puffs as needed for asthma (last use about 1 month ago)  Over the counter/dose/reason  - loratadine 10mg daily in the spring and fall for allergies  - acetaminophen 500mg as needed for aches/pains after working out at the gym (last use this morning)  Herbs/supplements/dose/reason  - none  Other: |
| Immunizations | X Tetanus  X Flu  X Hepatitis   - Pneumovax - HPV - Other |
| Tobacco products:   - Cigarettes - Cigar - Pipe - Chew - E-cigarettes | X Never   - Past- year started/year quit - Current   - Quantity   - # of years |
| Alcohol  X Beer   - Wine - Liquor - Other | - Never - Past- year started/year quit   X Current   - - Quantity: 2-3 beers on the weekends at parties   - # of years: 1-2 |
| Drugs   - Weed - Cocaine - Heroin - Meth - Other - IV - Inhalants - Other | X Never   - Past- year started/year quit - Current   - Quantity - # of years |
| Diet (describe) | Typical diet includes pizza, sandwiches, hamburgers for lunch and a healthy dinner at night that my Mom cooks |
| Exercise (describe) | Attend the gym 2-3 times per week and like to stay active by running and biking |
| List any other important social history or information important to this case | None |
| Family history |  |
| Mother, Father, Siblings, Grandparents, and other significant findings. | Mother: alive and healthy, has diabetes type 2  Father: alive and healthy, has Gastroesophageal Reflux Disease (GERD)  Sister: older sister also has GERD |
|  |  |
| Physical Exam-  Vitals: temp 100.8, BP 135/82, HR 85, RR 14, pulse ox 99% on RA BMI 21  General appearance: sitting still in the chair (or on exam table), holding your abdomen, appear somewhat uncomfortable  Abdominal exam:   - Normal to inspection - Bowel sounds normal - Mild tenderness to light palpation everywhere, worse in the right lower quadrant - Moderate tenderness to deep palpation in the right lower quadrant - No rebound or guarding (feels the same when the student presses down as when they let go; you allow them to do the exam and tense up a little bit, but not too much) - Tenderness is worse at McBurney’s point (about 2 inches from the anterior superior iliac spine (ASIS) on a line from the ASIS to the umbilicus) - Positive Rovsing’s Sign: when pressure is applied to your left lower quadrant, you feel the pain in the right lower quadrant - Positive Psoas Sign: if the student asks you to lay down and lift your right leg up against resistance, you feel pain in the right lower quadrant (not in the leg). Alternatively, the student may have you lie on your left side and pull your right leg back, which would also cause pain in the right lower quadrant. - Negative heel jar test: if the student taps forcefully on your foot while you are lying down, you do not have significant abdominal pain | |
| PHYSICAL EXAM FINDINGS |  |
| 1. Written in layman’s terms |  |
| 1. General appearance- affect, appearance, position of patient at opening (i.e. sitting, laying down, holding abdomen etc.) |  |
| 1. Vital signs |  |
| 1. Specific findings and affect |  |
| 1. Response to certain physical movements |  |
|  |  |
| DIAGNOSIS AND DIFFERENTIAL |  |
| Diagnosis with support from positive and negative history and PE findings | This patient has acute appendicitis. This is the most likely diagnosis based on the patient’s report of worsening abdominal pain that began around the umbilicus and settled into the right lower quadrant with associated anorexia, nausea, and fever. The physical exam findings of tenderness at McBurney’s point as well as the positive Rovsing’s and Psoas Signs are also suggestive of acute appendicitis. |
| Differential with support from positive and negative history and PE findings | This patient has acute appendicitis. Other diagnoses to consider include Crohn’s Disease, peptic ulcer disease, kidney stone, and acute pancreatitis. |
|  |  |
| MANAGEMENT OR DIAGNOSTIC PLAN | Appropriate evaluation in this case would include ordering a complete blood count, complete metabolic panel, urinalysis, and CT of the abdomen and pelvis. You may also consider ordering a serum lipase if acute pancreatitis is high on the differential. |
|  |  |
| PROFESSIONALISM ISSUES OR CHALLENGES: | None |

*Case 2: Acute Cholecystitis*

Date: July 29, 2019

Primary Case Author: Donna Williams, MD

Secondary Case Author: Joel Bruggen, MD

Standardized Patient Educator: Donna Williams, MD and Joel Bruggen, MD

Name of Case: Acute cholecystitis

Name of educational and or assessment activity: GI Simulated Clinic

Patient Name: Amy/Adam Morgan

Chief Complaint: “my stomach hurts, and I’ve had nausea and vomiting.”

Most likely Diagnosis and Differential with rationale from history and/or physical exam: This patient has acute cholecystitis. Acute cholecystitis is most likely in this case due to the patient’s demographics, pattern of pain with radiation to the shoulder, and physical exam findings including the positive Murphy’s sign. Other diagnoses to consider include peptic ulcer disease, acute pancreatitis, ascending cholangitis, small bowel obstruction, and kidney stone.

Challenge question: None

Domains: Check all that apply

- Professionalism

X Communication and Interpersonal skills

X Medical History

X Physical exam

- Shared Decision Making
- Patient Education

X Clinical Reasoning

- Documentation
- Handoff
- Presentation
- Other:

Type and level of learner: First or second year medical students

Case Objectives:

1. Medical History:
   1. Use hypothesis-driven data gathering to identify key or distinguishing features of a patient’s clinical presentation in a time-limited encounter.
2. Physical Exam:
   1. Interpret physical exam (PE) findings to further characterize a patient’s problem representation.
3. Clinical Reasoning:
   1. Compare and contrast a patient’s problem representation with one’s illness scripts to formulate an appropriate, prioritized differential diagnosis.
   2. Suggest appropriate diagnostic testing based on one’s differential diagnosis.

| SETTING: outpatient, in patient, ED, home, nursing home, rehab, group etc. | Emergency Department |
| --- | --- |
| PATIENT PROFILE: Information about the “patient” that helps select an SP and helps the learner get an understanding of them as a person. SP will know more information about the patient than learner will ever ask but allows SP to portray a fully developed patient personality. If none of the items below are particulars for the case please write “all may be used.” | |
| Age range | 40 - 50 years |
| Religious/spiritual background | Any |
| Sex (e.g., male, female, intersex, transwoman, transman) | Any |
| Sexual Orientation (e.g., heterosexual, lesbian, gay, bisexual, pansexual, queer, asexual) | Any |
| Gender expression (e.g., man, woman, gender queer) | Any |
| Race/ethnicity: | Any |
| Physical description (e.g., BMI, height range) | Overweight to obese; ideal BMI around 30 |
| Physical limitations | None |
| Patient appearance (e.g., disheveled, hospital gown, business casual, casual) | Hospital gown |
| Moulage + location (e.g., none, bruises, scars, body piercing, tattoos) | None |
| Affect (e.g., pleasant, cooperative) | Pleasant, cooperative |
| Family group (e.g., who is family, who they live with) | You live with your spouse and two sons, ages 15 and 17 |
| Education | Graduated college with a degree in communications. |
| Level of health literacy | Average |
| Employment, if any - present and past, noting any current stresses | You work in human resources for a local hospital. |
| Home/homeless - type of dwelling, number of stories, owned or rented | You live in a single family home with your spouse and children. |
| Financial situation- any current stresses | No current stresses. |
| Insurance Status (e.g., un/under/insured, public/private, HMO/PPO) | You are insured through your job. |
| Habits (i.e., diet, exercise, caffeine, smoking, alcohol, drugs) | Diet: You eat out a lot and don’t do a lot of cooking at home; you know your diet isn’t great.  Exercise: You tend to your garden and walk your dog, but do not have a specific exercise routine.  Tobacco: smoke cigarettes, 1 pack per day for the last 25 years  Alcohol: none  Drugs: none |
| Activities (i.e., hobbies, sports, clubs, friends) | You enjoy gardening and spending time with your family and your dog. |
| Typical day - what is the usual daily routine | On a typical day, you go to work from 9am to 5pm, then meet your family at home or after school activities, eat dinner, watch TV, and go to bed. |

| CASE INFORMATION | |
| --- | --- |
| Chief Concern: What the patient will say when greeted by the student. The patient’s primary reason for seeking medical care often stated in his/own words. | My stomach is really hurting me; it started right after I ate breakfast today and it’s so bad that I thought I should come in. |
| Additional Concerns: Other, if any, concerns the patient has today (i.e., symptoms, requests, expectations, etc.) that will become part of set agenda. | None |
|  | |
| THE PATIENT STORY: The SP will be asked to tell their symptom story and the personal and emotion impact for each of their concerns. You will want to write this is the patient voice. The symptom story should be able to answer this question: “Tell me more about [chief concern/additional concern], starting at the beginning and bringing me up to now.”  The personal context should be able to answer questions concerning the broader personal/psychosocial context of symptoms, especially the patient beliefs/attributions.  The emotional context should be able to ask how are you doing with this, how does this make you feel, how has this affected you emotionally? IMPACT: How has this affected your life? How has this been for your family? | I was feeling fine until this morning. I went out to breakfast at a local diner with an old friend. I had a big breakfast of scrambled eggs with cheese and bacon and a couple pancakes with syrup. I went home, and about an hour later started feeling this terrible pain in my stomach. I remember my mother had pan like this when she had her heart attack a while back, which got me worried enough to come in to the ER. I really hope it’s just food poisoning though. |
| HISTORY OF PRESENT ILLNESS: Although some of the HPI will be given in the patient’s symptom story, the learners will expand the story during the direct question section. Below describe the detailed history, usually about the chief concern, which the student must develop in order to make a useful assessment of the problem: | |
|  | |
| Onset (when; gradual or sudden) | Started today after breakfast, about 4 hours ago, and is getting worse |
| Setting (what was going on or where was patient when symptoms first noticed?) | Noticed after eating a large breakfast |
| Duration (how long) | About 4 hours |
| Time relationships (frequency, constant or intermittent) | Pain is constant |
| Location | The pain is under my rib cage on the right side |
| Radiation | The pain seems to go into my right shoulder too |
| Quality | sharp |
| Amount | Severity initially was 5 out of 10, and got up to an 8 out of 10. Now it’s back down to a 5 out of 10. |
| Aggravated by what | Nothing. I haven’t tried to eat or drink anything since this started; I’m afraid to. |
| Relieved by what | Nothing. I tried taking some Tums, but that didn’t help at all. Changing positions doesn’t seem to help either. |
| Associated with what | - Nausea: I started feeling nauseous around the same time as the pain started. It’s starting to feel a little better now, but it’s not gone.  - Vomiting: I vomited twice today since breakfast. First, it just looked like food that came up, but the second time it was clear liquid with some green in it. (No blood or dark material) |
| Attitude (what does the patient think is the problem, and how does he/she feel about it) | I wonder if I got food poisoning from breakfast, but my friend seems to be fine. I have had pain in my stomach after eating before (a few times over the last 6 months or so), but it’s never been this bad. In the past I’ve taken Tums and it’s seemed to help, but didn’t do anything this time. |
| Overall course | Worsening since pain began after breakfast, just now starting to back off a bit. |
| REVIEW OF SYSTEMS: Significant positives and negatives | |
| Fever/Chills | Over the last couple hours I’ve started to feel feverish and have some chills, but I haven’t checked my temperature. |
| Vomiting/Diarrhea/Constipation/Stool Changes | I typically have one formed stool per day, and had one regular looking bowel movement this morning. |
| GYN (if a woman) | I’ve had irregular periods all my life, and since I got my IUD a couple years ago, I have some spotting every 5 or 6 weeks, but no true periods. |
|  | |
| Past medical history | Diabetes type 2, high cholesterol |
| Medication allergies (Name and reaction) | None |
| Environmental allergies (Name and reaction) | None |
| Illnesses | None |
| Vaccinations | I’ve had all my regular vaccines, as far as I know |
| Surgeries | None |
| Accidents/ injuries/ trauma | None |
| Hospitalization | None |
|  | |
| Inclusive sexual and reproductive history | |
| Sexual practices  Sexual partners  Protection: Use of safer sex practices  Use of birth control if appropriate  Risk of intimate partner violence | Heterosexual  One current sexual partner (spouse; two total lifetime partners)  Does not use barrier protection  Has IUD for contraception |
| Ob/GYN HISTORY (if woman) | Age of onset of menses 14  Age of menopause N/A  Number of pregnancies 2  Number of live births 2  Number of miscarriages 0  Number of abortions 0 |
| Medications | Prescription/dose/reason  - Metformin, 1000mg twice a day for diabetes  - Atorvastatin 40mg every night for high cholesterol  Over the counter/dose/reason  - none  Herbs/supplements/dose/reason  - none  Other: |
| Immunizations | X Tetanus  X Flu  X Hepatitis   - Pneumovax - HPV - Other |
| Tobacco products:  X Cigarettes   - Cigar - Pipe - Chew - E-cigarettes | - Never - Past- year started/year quit   X Current   - - Quantity: 1 pack per day   - # of years: 25 |
| Alcohol   - Beer - Wine - Liquor - Other | X Never   - Past- year started/year quit - Current   - Quantity:   - # of years: |
| Drugs   - Weed - Cocaine - Heroin - Meth - Other - IV - Inhalants - Other | X Never   - Past- year started/year quit - Current   - Quantity - # of years |
| Diet (describe) | You eat out a lot and don’t do a lot of cooking at home; you know your diet isn’t great. |
| Exercise (describe) | None other than walking the dog and working in the garden |
| List any other important social history or information important to this case | None |
| Family history |  |
| Mother, Father, Siblings, Grandparents, and other significant findings. | Mother: alive, has diabetes type 2 and high cholesterol; had a heart attack at age 60  Father: alive with hypertension and diabetes  Children: alive and healthy |
|  |  |
| Physical Exam-  Vitals: temp 100.2, BP 155/80, HR 95, RR 14, pulse ox 98% on RA BMI 30  Gen: sitting in the chair trying to sit as still as possible, appear somewhat uncomfortable  Abdominal exam:   - Normal to inspection - Bowel sounds decreased - Moderate tenderness to light and deep palpation everywhere, but worse in the right upper quadrant - Positive for rebound and guarding (pain worsens when the student releases pressure after deep palpation; you tense up during the exam and make it difficult for them to fully examine your abdomen) - Positive Murphy’s Sign – when the student presses in under your right rib cage (right upper quadrant) and asks you to take a deep breath, you stop breathing during inspiration due to severe pain - Positive heel jar test: if the student taps forcefully on your foot while you are lying down, you have significant abdominal pain (not pain in the foot) - All other potential maneuvers the student may try do not cause increased abdominal pain. | |
| PHYSICAL EXAM FINDINGS |  |
| 1. Written in layman’s terms |  |
| 1. General appearance- affect, appearance, position of patient at opening (i.e. sitting, laying down, holding abdomen etc.) |  |
| 1. Vital signs |  |
| 1. Specific findings and affect |  |
| 1. Response to certain physical movements |  |
|  |  |
| DIAGNOSIS AND DIFFERENTIAL |  |
| Diagnosis with support from positive and negative history and PE findings | This patient has acute cholecystitis. Acute cholecystitis is most likely in this case due to the patient’s demographics, pattern of pain with radiation to the shoulder, and physical exam findings including the positive Murphy’s sign. |
| Differential with support from positive and negative history and PE findings | This patient has acute cholecystitis. Other diagnoses to consider include peptic ulcer disease, acute pancreatitis, ascending cholangitis, small bowel obstruction, and kidney stone. |
|  |  |
| MANAGEMENT OR DIAGNOSTIC PLAN | Appropriate evaluation in this case would include ordering a complete blood count, complete metabolic panel, urinalysis, and CT of the abdomen and pelvis. You may also consider ordering an abdominal ultrasound (if you are not considering small bowel obstruction), serum lipase if acute pancreatitis is high on the differential, and a pregnancy test if the patient is a woman. |
|  |  |
| PROFESSIONALISM ISSUES OR CHALLENGES: | None |

*Case 3: Inflammatory Bowel Disease*

Date: July 29, 2019

Primary Case Author: Donna Williams, MD

Secondary Case Author: Joel Bruggen, MD

Standardized Patient Educator: Donna Williams, MD and Joel Bruggen, MD

Name of Case: Inflammatory Bowel Disease (IBD)

Name of educational and or assessment activity: GI Simulated Clinic

Patient Name: Thomas/Tina Reese

Chief Complaint: “I’ve been having bloody diarrhea.”

Most likely Diagnosis and Differential with rationale from history and/or physical exam: This patient has inflammatory bowel disease. Inflammatory bowel disease is most likely in this case due to the patient’s age, weight loss, and symptoms of progressive diarrhea with blood in the absence of known exposure to causative organisms. Other diagnoses to consider include infectious gastroenteritis.

Challenge question: None

Domains: Check all that apply

- Professionalism

X Communication and Interpersonal skills

X Medical History

X Physical exam

- Shared Decision Making
- Patient Education

X Clinical Reasoning

- Documentation
- Handoff
- Presentation
- Other:

Type and level of learner: First or second year medical students

Case Objectives:

1. Medical History:
   1. Use hypothesis-driven data gathering to identify key or distinguishing features of a patient’s clinical presentation in a time-limited encounter.
2. Physical Exam:
   1. Interpret physical exam (PE) findings to further characterize a patient’s problem representation.
3. Clinical Reasoning:
   1. Compare and contrast a patient’s problem representation with one’s illness scripts to formulate an appropriate, prioritized differential diagnosis.
   2. Suggest appropriate diagnostic testing based on one’s differential diagnosis.

| SETTING: outpatient, in patient, ED, home, nursing home, rehab, group etc. | Internal medicine or family medicine clinic |
| --- | --- |
| PATIENT PROFILE: Information about the “patient” that helps select an SP and helps the learner get an understanding of them as a person. SP will know more information about the patient than learner will ever ask but allows SP to portray a fully developed patient personality. If none of the items below are particulars for the case please write “all may be used.” | |
| Age range | 24 - 30 years |
| Religious/spiritual background | Any |
| Sex (e.g., male, female, intersex, transwoman, transman) | Any |
| Sexual Orientation (e.g., heterosexual, lesbian, gay, bisexual, pansexual, queer, asexual) | Lesbian (if woman), gay (if man) |
| Gender expression (e.g., man, woman, gender queer) | Any |
| Race/ethnicity: | Any |
| Physical description (e.g., BMI, height range) | Average BMI |
| Physical limitations | None |
| Patient appearance (e.g., disheveled, hospital gown, business casual, casual) | Dressed in street clothes or hospital gown |
| Moulage + location (e.g., none, bruises, scars, body piercing, tattoos) | None |
| Affect (e.g., pleasant, cooperative) | Pleasant, cooperative |
| Family group (e.g., who is family, who they live with) | You live in an apartment with your significant other (same sex partner) |
| Education | Graduated college with a degree in engineering |
| Level of health literacy | Average to high |
| Employment, if any - present and past, noting any current stresses | You work as an engineer for a medical device company. |
| Home/homeless - type of dwelling, number of stories, owned or rented | You live with your significant other in a rented apartment. |
| Financial situation- any current stresses | None |
| Insurance Status (e.g., un/under/insured, public/private, HMO/PPO) | You have insurance through your job. |
| Habits (i.e., diet, exercise, caffeine, smoking, alcohol, drugs) | Diet: You eat a balanced diet of meat, vegetables, and starches.  Exercise: You exercise regularly, usually by running.  Tobacco: none (never smoker)  Alcohol: you drink beer on the weekends if you go out with friends, usually 2 or 3 beers per night on these occasions.  Drugs: none (never) |
| Activities (i.e., hobbies, sports, clubs, friends) | You like to watch movies. |
| Typical day - what is the usual daily routine | You typically get up early to go for a run before work. You work 8-10 hour days, then come home and have dinner and relax. |

| CASE INFORMATION | |
| --- | --- |
| Chief Concern: What the patient will say when greeted by the student. The patient’s primary reason for seeking medical care often stated in his/own words. | I’ve been having this awful diarrhea, and now it has blood in it so I’m starting to get worried. |
| Additional Concerns: Other, if any, concerns the patient has today (i.e., symptoms, requests, expectations, etc.) that will become part of set agenda. | None |
|  | |
| THE PATIENT STORY: The SP will be asked to tell their symptom story and the personal and emotion impact for each of their concerns. You will want to write this is the patient voice. The symptom story should be able to answer this question: “Tell me more about [chief concern/additional concern], starting at the beginning and bringing me up to now.”  The personal context should be able to answer questions concerning the broader personal/psychosocial context of symptoms, especially the patient beliefs/attributions.  The emotional context should be able to ask how are you doing with this, how does this make you feel, how has this affected you emotionally? IMPACT: How has this affected your life? How has this been for your family? | I’ve always had about 2 bowel movements per day, but for the last 2 months or so I seem to have more. First I was having about 4 loose bowel movements a day, but now it’s happening 8 or 10 times a day. For the last 3 weeks, I’ve noticed some blood in the stool too. It seems to be bright red, and mixed in with the stool.  I’ve also had some stomach cramping, but no other pain. Sometimes I wake up in the middle of the night to have to have a bowel movement; that never used to happen.  I can’t remember doing anything different before all this started. I haven’t traveled anywhere, eaten anything weird, or been around people who have been sick. |
| HISTORY OF PRESENT ILLNESS: Although some of the HPI will be given in the patient’s symptom story, the learners will expand the story during the direct question section. Below describe the detailed history, usually about the chief concern, which the student must develop in order to make a useful assessment of the problem: | |
|  | |
| Onset (when; gradual or sudden) | Increased stools started gradually about 2 months ago |
| Setting (what was going on or where was patient when symptoms first noticed?) | Nothing notable |
| Duration (how long) | About 2 months |
| Time relationships (frequency, constant or intermittent) | Diarrhea has been worsening since it began |
| Location | N/A |
| Radiation | N/A |
| Quality | Stools are loose and brown, now with blood mixed in |
| Amount | 8 to 10 times per day, moderate to large amount of stool each episode |
| Aggravated by what | Nothing |
| Relieved by what | Nothing. I tried taking some immodium, but it didn’t help. I tried changing my diet by taking out dairy products, but that didn’t seem to make any difference either. |
| Associated with what | - abdominal cramping: happens occasionally, relieve by having a bowel movement |
| Attitude (what does the patient think is the problem, and how does he/she feel about it) | Aside from having severe diarrhea with a little bleeding after a trip to Mexico 2 years ago that resolved, I’ve never had anything like this before. |
| Overall course | Progressively worsening since the diarrhea began |
| REVIEW OF SYSTEMS: Significant positives and negatives | |
| Fever/Chills | None |
| Weight changes | I’ve lost about 10 pounds since this started 2 months ago. I haven’t been trying to lose weight. |
| Fatigue | I feel like I have less energy than usual, and have been skipping my morning run most days because of this. But I am still able to go to work and go about the rest of my day. |
| GYN (if a woman) | I’ve always had regular periods lasting 3-5 days with moderate flow every 28 days. My last period was 2 weeks ago. |
|  | |
| Past medical history | None |
| Medication allergies (Name and reaction) | None |
| Environmental allergies (Name and reaction) | None |
| Illnesses | I had appendicitis when I was 12, otherwise have been pretty healthy. |
| Vaccinations | I’ve had all my regular vaccines, as far as I know |
| Surgeries | Appendectomy at age 12 |
| Accidents/ injuries/ trauma | None |
| Hospitalization | None |
|  | |
| Inclusive sexual and reproductive history | |
| Sexual practices  Sexual partners  Protection: Use of safer sex practices  Use of birth control if appropriate  Risk of intimate partner violence | Homosexual  One current sexual partner (2 total lifetime partners)  Uses condoms (if man)  You and your partner have been tested for HIV and other STIs in the past, most recently 6 months ago. These tests have always been negative. |
| Ob/GYN HISTORY (if woman) | Age of onset of menses 12  Age of menopause N/A  Number of pregnancies 0  Number of live births 0  Number of miscarriages 0  Number of abortions 0 |
| Medications | Prescription/dose/reason  - none  Over the counter/dose/reason  - none regularly, although you tried a couple doses of over the counter immodium without getting any relief  Herbs/supplements/dose/reason  - none  Other: |
| Immunizations | X Tetanus   - Flu - Hepatitis - Pneumovax - HPV - Other |
| Tobacco products:   - Cigarettes - Cigar - Pipe - Chew - E-cigarettes | X Never   - Past- year started/year quit - Current   - Quantity: 1 pack per day   - # of years: 30 |
| Alcohol  X Beer   - Wine - Liquor - Other | - Never - Past- year started/year quit   X Current   - - Quantity: 2-3 bottles on the weekends if going out with friends   - # of years: 2-4 years |
| Drugs   - Weed - Cocaine - Heroin - Meth - Other - IV - Inhalants - Other | X Never   - Past- year started/year quit - Current   - Quantity - # of years |
| Diet (describe) | You eat a balanced diet of meat, vegetables, and starches. |
| Exercise (describe) | You run regularly for exercise. |
| List any other important social history or information important to this case | None |
| Family history |  |
| Mother, Father, Siblings, Grandparents, and other significant findings. | Mother: alive, has lupus which is well controlled with medications  Father: alive with hypothyroidism  Sister: alive and healthy |
|  |  |
| Physical Exam-  Vitals: temp 98.8, BP 125/80, HR 75, RR 14, pulse ox 100% on RA  General appearance: no acute distress, you appear comfortable  HEENT: conjunctival rims pale (make card for SP to present to students if they examine the conjunctiva)  Abdominal exam:   - Normal to inspection - Bowel sounds normal - Slight tenderness to deep palpation of left lower quadrant - No rebound or guarding (feels the same when the student presses down as when they let go; you allow them to do the exam and tense up a little bit, but not too much) - Liver normal, spleen non-palpable - All other potential maneuvers the student may try do not cause increased abdominal pain. - Rectal exam with gross blood; no internal or external hemorrhoids noted. (make card for SP to present to students if they ask to perform a rectal exam) | |
| PHYSICAL EXAM FINDINGS |  |
| 1. Written in layman’s terms |  |
| 1. General appearance- affect, appearance, position of patient at opening (i.e. sitting, laying down, holding abdomen etc.) |  |
| 1. Vital signs |  |
| 1. Specific findings and affect |  |
| 1. Response to certain physical movements |  |
|  |  |
| DIAGNOSIS AND DIFFERENTIAL |  |
| Diagnosis with support from positive and negative history and PE findings | This patient has inflammatory bowel disease. Inflammatory bowel disease is most likely in this case due to the patient’s age, weight loss, and symptoms of progressive diarrhea with blood in the absence of known exposure to causative organisms. |
| Differential with support from positive and negative history and PE findings | This patient has inflammatory bowel disease. Other diagnoses to consider include infectious gastroenteritis. |
|  |  |
| MANAGEMENT OR DIAGNOSTIC PLAN | Appropriate evaluation in this case would include ordering a complete blood count, complete metabolic panel, and stool culture. The next step would include performing a colonoscopy. |
|  |  |
| PROFESSIONALISM ISSUES OR CHALLENGES: | None |

*Case 4: Pancreatic Cancer*

Date: July 29, 2019

Primary Case Author: Donna Williams, MD

Secondary Case Author: Joel Bruggen, MD

Standardized Patient Educator: Donna Williams, MD and Joel Bruggen, MD

Name of Case: Pancreatic Cancer

Name of educational and or assessment activity: GI Simulated Clinic

Patient Name: Jane/Joe Anderson

Chief Complaint: “I’ve been losing weight, and my skin is yellow and itchy.”

Most likely Diagnosis and Differential with rationale from history and/or physical exam: This patient has pancreatic cancer. Pancreatic cancer is most likely in this case due to the patient’s age, weight loss, and symptoms of painless jaundice with jaundice found on exam. Other diagnoses to consider include hepatitis, cirrhosis, gastric cancer, and cholangiocarcinoma.

Challenge question: None

Domains: Check all that apply

- Professionalism

X Communication and Interpersonal skills

X Medical History

X Physical exam

- Shared Decision Making
- Patient Education

X Clinical Reasoning

- Documentation
- Handoff
- Presentation
- Other:

Type and level of learner: First or second year medical students

Case Objectives:

1. Medical History:
   1. Use hypothesis-driven data gathering to identify key or distinguishing features of a patient’s clinical presentation in a time-limited encounter.
2. Physical Exam:
   1. Interpret physical exam (PE) findings to further characterize a patient’s problem representation.
3. Clinical Reasoning:
   1. Compare and contrast a patient’s problem representation with one’s illness scripts to formulate an appropriate, prioritized differential diagnosis.
   2. Suggest appropriate diagnostic testing based on one’s differential diagnosis.

| SETTING: outpatient, in patient, ED, home, nursing home, rehab, group etc. | Internal medicine or family medicine clinic |
| --- | --- |
| PATIENT PROFILE: Information about the “patient” that helps select an SP and helps the learner get an understanding of them as a person. SP will know more information about the patient than learner will ever ask but allows SP to portray a fully developed patient personality. If none of the items below are particulars for the case please write “all may be used.” | |
| Age range | 55 - 70 years |
| Religious/spiritual background | Any |
| Sex (e.g., male, female, intersex, transwoman, transman) | Any |
| Sexual Orientation (e.g., heterosexual, lesbian, gay, bisexual, pansexual, queer, asexual) | Heterosexual |
| Gender expression (e.g., man, woman, gender queer) | Any |
| Race/ethnicity: | Any |
| Physical description (e.g., BMI, height range) | Average BMI |
| Physical limitations | None |
| Patient appearance (e.g., disheveled, hospital gown, business casual, casual) | Dressed in street clothes or hospital gown |
| Moulage + location (e.g., none, bruises, scars, body piercing, tattoos) | None |
| Affect (e.g., pleasant, cooperative) | Pleasant, cooperative |
| Family group (e.g., who is family, who they live with) | You live with your spouse and have 3 adult children who live in the area (ages 26, 28, and 31). |
| Education | Graduated high school |
| Level of health literacy | Average |
| Employment, if any - present and past, noting any current stresses | You work as a bank teller at a local bank |
| Home/homeless - type of dwelling, number of stories, owned or rented | You live with your spouse in a single family home that you own. |
| Financial situation- any current stresses | None |
| Insurance Status (e.g., un/under/insured, public/private, HMO/PPO) | You have insurance through your job. |
| Habits (i.e., diet, exercise, caffeine, smoking, alcohol, drugs) | Diet: You eat a balanced diet of meat, vegetables, and starches.  Exercise: None  Tobacco: You have smoked one pack of cigarettes per day for 40 years and continue to smoke.  Alcohol: You drink wine with dinner occasionally, about 4-5 glasses per week. You haven’t had much lately though as you haven’t really felt like dinking it.  Drugs: none (never) |
| Activities (i.e., hobbies, sports, clubs, friends) | You like to read mystery novels, travel, and spend time with your spouse and children. |
| Typical day - what is the usual daily routine | You typically go to work for about 8 hours, come home and have dinner with your spouse, and relax by reading or watching TV before bedtime. |

| CASE INFORMATION | |
| --- | --- |
| Chief Concern: What the patient will say when greeted by the student. The patient’s primary reason for seeking medical care often stated in his/own words. | I’ve been losing weight without trying, and have been itchy. My daughter says my skin looks yellow too. |
| Additional Concerns: Other, if any, concerns the patient has today (i.e., symptoms, requests, expectations, etc.) that will become part of set agenda. | None |
|  | |
| THE PATIENT STORY: The SP will be asked to tell their symptom story and the personal and emotion impact for each of their concerns. You will want to write this is the patient voice. The symptom story should be able to answer this question: “Tell me more about [chief concern/additional concern], starting at the beginning and bringing me up to now.”  The personal context should be able to answer questions concerning the broader personal/psychosocial context of symptoms, especially the patient beliefs/attributions.  The emotional context should be able to ask how are you doing with this, how does this make you feel, how has this affected you emotionally? IMPACT: How has this affected your life? How has this been for your family? | Over the past 3 months, I’ve lost about 20 pounds. I haven’t been trying to lose weight, but I haven’t felt like eating as much as usual either. For the past month, I’ve been itchy all over. I’ve been using all the same soaps and laundry detergents I always use and haven’t changed anything.  Last week, my daughter came over for a visit and she told me that I looked yellow and that I should make an appointment to see my doctor, so here I am to get checked out. |
| HISTORY OF PRESENT ILLNESS: Although some of the HPI will be given in the patient’s symptom story, the learners will expand the story during the direct question section. Below describe the detailed history, usually about the chief concern, which the student must develop in order to make a useful assessment of the problem: | |
|  | |
| Onset (when; gradual or sudden) | Weight loss: 3 months, gradual  Itchy skin: 1 month, gradual |
| Setting (what was going on or where was patient when symptoms first noticed?) | Nothing notable |
| Duration (how long) | See above |
| Time relationships (frequency, constant or intermittent) | Constant |
| Location | Itchy skin is everywhere on my body, no one spot in particular |
| Radiation | N/A |
| Quality | N/A |
| Amount | I’ve lost about 20 pounds |
| Aggravated by what | The itchiness seems to get worse the more I scratch at it, but otherwise nothing in particular seems to bother it more. |
| Relieved by what | I tried some taking some Benadryl and using some Benadryl cream, but neither of them provided any relief. |
| Associated with what | I haven’t really felt like eating much in the last couple months. I eat a few bites at each meal, but just don’t feel hungry and don’t want to eat.  My daughter told me that my skin looks yellow when I saw her last week. Once she mentioned, I looked closely in the mirror and thought that my eyes and skin do look a little yellow, but I hadn’t noticed it before she mentioned it. |
| Attitude (what does the patient think is the problem, and how does he/she feel about it) | I’ve never had any of these problems before; I hope it’s not something serious. |
| Overall course | Progressive weight loss and itchiness |
| REVIEW OF SYSTEMS: Significant positives and negatives | |
| Fever/Chills/Night sweats | None |
| Weight changes | I’ve lost about 20 pounds since this started 3 months ago. |
| Fatigue | I feel a little more tired than usual, but nothing that stops me from doing what I usually do. |
| Skin | No rashes |
| GYN (if a woman) | I had regular periods all my life, until I went through menopause about 4 years ago (range: 4-15 years ago, based on patient age) |
|  | |
| Past medical history | High blood pressure, diagnosed 10 years ago, well controlled |
| Medication allergies (Name and reaction) | None |
| Environmental allergies (Name and reaction) | None |
| Illnesses | None |
| Vaccinations | I’ve had all my regular vaccines, as far as I know |
| Surgeries | None if woman; vasectomy if man |
| Accidents/ injuries/ trauma | None |
| Hospitalization | None |
|  | |
| Inclusive sexual and reproductive history | |
| Sexual practices  Sexual partners  Protection: Use of safer sex practices  Use of birth control if appropriate  Risk of intimate partner violence | Heterosexual  One current sexual partner (1 total lifetime partners)  No barrier protection  Vasectomy (in self if man, spouse if woman) for contraception |
| Ob/GYN HISTORY (if woman) | Age of onset of menses 14  Age of menopause 51  Number of pregnancies 4  Number of live births 3  Number of miscarriages 1  Number of abortions 0 |
| Medications | Prescription/dose/reason  - Hydrochlorothiazide 25mg daily for high blood pressure  Over the counter/dose/reason  - none regularly, although you tried a couple doses of over the counter Benadryl without getting any relief  Herbs/supplements/dose/reason  - none  Other: |
| Immunizations | X Tetanus   - Flu - Hepatitis - Pneumovax - HPV - Other |
| Tobacco products:  X Cigarettes   - Cigar - Pipe - Chew - E-cigarettes | - Never - Past- year started/year quit   X Current   - - Quantity: 1 pack per day   - # of years: 40 |
| Alcohol   - Beer   X Wine   - Liquor - Other | - Never - Past- year started/year quit   X Current   - - Quantity: 1 drink with dinner, about 5 nights per week   - # of years: 20 |
| Drugs   - Weed - Cocaine - Heroin - Meth - Other - IV - Inhalants - Other | X Never   - Past- year started/year quit - Current   - Quantity - # of years |
| Diet (describe) | You eat a balanced diet of meat, vegetables, and starches. |
| Exercise (describe) | None |
| List any other important social history or information important to this case | None |
| Family history |  |
| Mother, Father, Siblings, Grandparents, and other significant findings. | Mother: died from lung cancer at age 70 (she was a smoker)  Father: died from liver disease at age 66 (you don’t know what caused this, but he did drink at least a moderate amount of alcohol that you know about)  Brother: alive with high blood pressure  Children: alive and healthy |
|  |  |
| Physical Exam-  Vitals: temp 99.5, BP 145/84, HR 90, RR 14, pulse ox 99% on RA  General appearance: no distress, appear comfortable  HEENT: scleral icterus (make card for SP to present to students if they examine the sclera)  Skin: jaundice and excoriations (make card for SP to present to students if they examine the skin)  Abdominal exam: normal | |
| PHYSICAL EXAM FINDINGS |  |
| 1. Written in layman’s terms |  |
| 1. General appearance- affect, appearance, position of patient at opening (i.e. sitting, laying down, holding abdomen etc.) |  |
| 1. Vital signs |  |
| 1. Specific findings and affect |  |
| 1. Response to certain physical movements |  |
|  |  |
| DIAGNOSIS AND DIFFERENTIAL |  |
| Diagnosis with support from positive and negative history and PE findings | This patient has pancreatic cancer. Pancreatic cancer is most likely in this case due to the patient’s age, weight loss, and symptoms of painless jaundice with jaundice found on exam. |
| Differential with support from positive and negative history and PE findings | This patient has pancreatic cancer. Other diagnoses to consider include hepatitis, cirrhosis, gastric cancer, and cholangiocarcinoma. |
|  |  |
| MANAGEMENT OR DIAGNOSTIC PLAN | Appropriate evaluation in this case would include ordering a complete blood count, complete metabolic panel, and CT of the abdomen and pelvis. |
|  |  |
| PROFESSIONALISM ISSUES OR CHALLENGES: | None |
