# Supplementary material for: The GI Simulated Clinic: A Clinical Reasoning Exercise Supporting Medical Students' Basic and Clinical Science Integration
Source: MedEdPORTAL. 2020 Aug 5;16:10926. doi: 10.15766/mep_2374-8265.10926 (PMC7412764; doi:10.15766/mep_2374-8265.10926)
Supplement: Supplementary file 1 — SP Cases.docxPE Cards.docxLogistics.docxDoor Charts.docxWorksheets.docxDebrief.docxLearner Evaluation.docx [file mep_2374-8265.10926-s001.zip › B. PE Cards.docx]

**Thomas/Tina Reese Case Physical Exam (PE) cards**

**SP Instructions**

The following cards have been created for the physical exam (PE) portion of this case; these cards have been created only for PE maneuvers that SPs cannot act out.

Please follow these instructions for using the PE cards with each student group:

- **Keep these PE cards out of students’ view** until they “earn” the right to view them (by performing the applicable exam maneuver that corresponds to each card – see below for details).
- Give students the applicable card ***immediately following*** students’ performance of the specific physical exam maneuver required to “earn” each card.
- **Only give students the PE cards they “earn”** during the physical exam portion of the encounter; do not let them read/view other PE cards for which they did not perform the applicable maneuvers.

If students request to see PE cards they did not “earn,” tell the students that they are not allowed to do so, per instructions from the Course Director; but that they will be given additional information about this case after this learning event has concluded.

- **Collect all PE cards from students before they leave the exam room.** Students may write down the information from each PE card while they are in the room, but they must give the cards back to you before they leave.

**PE card label Exam maneuver required to “earn” this card**

EYES Inspection of inner eyelids: students will gently pull downward on the skin underneath your lower eyelids in order to inspect them.

MOUTH Inspection of mouth: students will ask you to open your mouth so that they can inspect your mouth, throat, and tongue (use of a light source to look into your mouth for this maneuver is not required for earning this PE card—give them the card even if they don’t use a light).

RECTUM Rectal exam: students will not actually perform this exam on you; if they mention or recommend doing a rectal exam, hand them this card.

| **EYES** | **EYES** | *Conjunctival pallor bilaterally* |
| --- | --- | --- |
| **MOUTH** | **MOUTH** | *Pallor of lips, tongue, and buccal mucosa* |

| **RECTUM** | **RECTUM** | *Bright red blood present;*  *No internal or external hemorrhoids noted* |
| --- | --- | --- |

**Jane/Joe Anderson Case Physical Exam (PE) cards**

**SP Instructions**

The following cards have been created for the physical exam (PE) portion of this case; these cards have been created only for PE maneuvers that SPs cannot act out.

Please follow these instructions for using the PE cards with each student group:

- **Keep these PE cards out of students’ view** until they “earn” the right to view them (by performing the applicable exam maneuver that corresponds to each card – see below for details).
- Give students the applicable card ***immediately following*** students’ performance of the specific physical exam maneuver required to “earn” each card.
- **Only give students the PE cards they “earn”** during the physical exam portion of the encounter; do not let them read/view other PE cards for which they did not perform the applicable maneuvers.

If students request to see PE cards they did not “earn,” tell the students that they are not allowed to do so, per instructions from the Course Director; but that they will be given additional information about this case after this learning event has concluded.

- **Collect all PE cards from students before they leave the exam room.** Students may write down the information from each PE card while they are in the room, but they must give the cards back to you before they leave.

**PE card label Exam maneuver required to “earn” this card**

EYES/SKIN Students would notice these findings as soon as they meet the patient; so give this card to the student when they enter the room and introduce themselves.

SKIN Inspection of skin: students will look directly at your skin, to look for skin changes, rashes, etc.

| **EYES/SKIN** | **EYES/SKIN** | *Yellow discoloration of skin throughout the body;*  *bilateral sclerae have yellow discoloration* |
| --- | --- | --- |
| **SKIN** | **SKIN** | *Excoriations present diffusely*  *on the patient’s arms and*  *legs, bilaterally* |
