# Supplementary material for: The GI Simulated Clinic: A Clinical Reasoning Exercise Supporting Medical Students' Basic and Clinical Science Integration
Source: MedEdPORTAL. 2020 Aug 5;16:10926. doi: 10.15766/mep_2374-8265.10926 (PMC7412764; doi:10.15766/mep_2374-8265.10926)
Supplement: Supplementary file 1 — SP Cases.docxPE Cards.docxLogistics.docxDoor Charts.docxWorksheets.docxDebrief.docxLearner Evaluation.docx [file mep_2374-8265.10926-s001.zip › C. Logistics.docx]

**Simulated GI Clinic: Logistics & Design Options**

**Timetable, day of event:**

| **Time** | **Activity** | | **Description** |
| --- | --- | --- | --- |
| 10 - 15 min prior to event start | Learners report to check-in area | | Staff/instructor distributes assigned small group schedules and worksheet packets to students as they check in (attendance may be recorded here as well, if desired). |
| 5 min prior to event start | Brief reminder of logistics for learners | | The instructor or other staff briefly reviews with students the logistics and their instructions for the activity. Small groups go to their first exam room. |
| 1:00p – 1:20p (20 min) | Patient encounter 1 | Patient encounter/data gathering: Student A | One student in each group interviews and examines the standardized patient (SP) in their assigned exam room (other students observe). |
| 1:20p – 1:30p (10 min) |  | Small group case discussion | Student small groups discuss the patient case, determine their differential diagnosis diagnostic plan, and complete the corresponding worksheet. |
| At 1:30p | Transition to next encounter | | Students move to their next assigned exam room. |
| 1:30p – 1:50p (20 min) | Patient encounter 2 | Patient encounter/data gathering: Student B | One student in each group interviews and examines the SP in their assigned exam room (other students observe). |
| 1:50p – 2:00p (10 min) |  | Small group case discussion | Student small groups discuss the patient case, determine their differential diagnosis diagnostic plan, and complete the corresponding worksheet. |
| At 2:00p | Transition to next encounter | | Students move to their next assigned exam room. |
| 2:00p – 2:20p (20 min) | Patient encounter 3 | Patient encounter/data gathering: Student C | One student in each group interviews and examines the SP in their assigned exam room (other students observe). |
| 2:20p – 2:30p (10 min) |  | Small group case discussion | Student small groups discuss the patient case, determine their differential diagnosis diagnostic plan, and complete the corresponding worksheet. |
| At 2:30p | Transition to next encounter | | Students move to their next assigned exam room. |
| 2:30p – 2:50p (20 min) | Patient encounter 4 | Patient encounter/data gathering: Student D | One student in each group interviews and examines the SP in their assigned exam room (other students observe). |
| 2:50p – 3:00p (10 min) |  | Small group case discussion | Student small groups discuss the patient case, determine their differential diagnosis diagnostic plan, and complete the corresponding worksheet. |
| 3:00p | Worksheet submission | | Students submit their team’s worksheets for review by the instructor. |
| TBD ^a^ | Debrief | | Students meet with the instructor(s) to go over the cases. |

*^a^ Scheduled according to students’ and instructor’s calendars.*

**Student group rotation schedule:**

The event duration can be adjusted according to the learner group size, one’s space needs and exam room availability, and one’s desired number of cases. The following is an example rotation schedule for an event designed for 12 student small groups, using 12 rooms, using all 4 cases, and running from 1pm to 3pm:

| **Encounter** | **Time** | **Case 1** | **Case 2** | **Case 3** | **Case 4** | **Case 1** | **Case 2** | **Case 3** | **Case 4** | **Case 1** | **Case 2** | **Case 3** | **Case 4** |
| --- | --- | --- | --- | --- | --- | --- | --- | --- | --- | --- | --- | --- | --- |
|  |  | Room 1 | Room 2 | Room 3 | Room 4 | Room 5 | Room 6 | Room 7 | Room 8 | Room 9 | Room 10 | Room 11 | Room 12 |
| 1^st^ case | 1p – 1:20p  (SP encounter) | Group 1 | Group 2 | Group 3 | Group 4 | Group 5 | Group 6 | Group 7 | Group 8 | Group 9 | Group 10 | Group 11 | Group 12 |
|  | 1:20p – 1:30p  (small group discussion) |  |  |  |  |  |  |  |  |  |  |  |  |
| 2^nd^ case | 1:30p – 1:50p  (SP encounter) | Group 12 | Group 1 | Group 2 | Group 3 | Group 4 | Group 5 | Group 6 | Group 7 | Group 8 | Group 9 | Group 10 | Group 11 |
|  | 1:50p – 2p  (small group discussion) |  |  |  |  |  |  |  |  |  |  |  |  |
| 3^rd^ case | 2p – 2:20p  (SP encounter) | Group 11 | Group 12 | Group 1 | Group 2 | Group 3 | Group 4 | Group 5 | Group 6 | Group 7 | Group 8 | Group 9 | Group 10 |
|  | 2:20p – 2:30p  (small group discussion) |  |  |  |  |  |  |  |  |  |  |  |  |
| 4^th^ case | 2:30p – 2:50p  (SP encounter) | Group 10 | Group 11 | Group 12 | Group 1 | Group 2 | Group 3 | Group 4 | Group 5 | Group 6 | Group 7 | Group 8 | Group 9 |
| Final small group discussion | | Student small groups complete and submit their worksheet packets. | | | | | | | | | | | |

*The following are examples of student schedule slips for 2 groups, based on the above rotation schedule (provided to students prior to event):*

| **Small group 1** | 1:00p - 1:30p | 1:30p - 2:00p | 2:00p - 2:30p | 2:30p - 3:00p |
| --- | --- | --- | --- | --- |
| Student A | Room 1  (Simmons) | Room 2  (Morton) | Room 3  (Reese) | Room 4  (Anderson) |
| Student B |  |  |  |  |
| Student C |  |  |  |  |
| Student D |  |  |  |  |
|  |  |  |  |  |
| **Small group 2** | 1:00p - 1:30p | 1:30p - 2:00p | 2:00p - 2:30p | 2:30p - 3:00p |
| Student E | Room 2  (Morton) | Room 3  (Reese) | Room 4  (Anderson) | Room 5  (Simmons) |
| Student F |  |  |  |  |
| Student G |  |  |  |  |
| Student H |  |  |  |  |

**Brief reminder of logistics for learners (immediately prior to event start):**

The following is an example of brief reminders/instructions for students just prior to the event start. This is helpful for ensuring learners understand the intended logistics and expectations of them during this event. These instructions can be tailored to the specific logistical model designed by the instructor.

- Carefully follow your group’s assigned schedule. Please stay on time for each encounter, so that the student group following yours can start on time. ^b^
- Review the simulated chart prior to entering each room.
- One student in your group should lead the data gathering for each encounter. You should take turns serving in this role, so that every student has an opportunity to lead at least 1 encounter by the end of the activity.
- A total of 20 minutes are allotted for you to gather data for each encounter, including both history taking and physical examination.
- Remember to apply the patient-centered communication and interpersonal techniques you have learned from the Clinical Skills Course.
- Following each SP encounter, your group will have an additional 10 minutes to discuss the case; use the worksheet provided for each case to document your differential diagnosis, diagnostic plan, and justification for both.
- After you have finished all 4 encounters, submit your group’s worksheet packet to the staff.

^b^ *Our simulation center has the capability for overhead intercom announcements, so we instructed students to listen for them throughout the activity, to facilitate staying on schedule*.

**Simulated GI Clinic: Design Options**

This activity can be tailored to the specific learning objectives desired by modifying its instructional design features, including:

Patient encounter skills practice options:

- History taking and communication skills practice, only
- History taking, communication, and limited physical examination (PE) skills practice: interpretation of simulated visual (e.g., printed or digital images) or audio findings (e.g., audio files available on a computer in the room)
- History taking, communication, and PE skills: PE maneuvers practice on standardized patients

Diagnostic reasoning skills practice options:

- Differential diagnosis, only
- Justified differential diagnosis (i.e., brief justification provided based on clinical findings from the history and/or PE)
- Differential diagnosis and suggested diagnostic studies (+/- justification)
- Differential diagnosis and suggested diagnostic studies (submitted), followed by interpretation of one or more study results and revised differential diagnosis

The timetable for patient encounters and small group case discussion durations should be adjusted accordingly, based on the design options selected. For example, if encounters consist of history taking and communication skills practice only, then the encounter times can be reduced to 10 minutes.

Similarly, students’ worksheets should be modified based on the diagnostic reasoning skills design chosen selected (see Appendix E for worksheet example).
