# Supplementary material for: The GI Simulated Clinic: A Clinical Reasoning Exercise Supporting Medical Students' Basic and Clinical Science Integration
Source: MedEdPORTAL. 2020 Aug 5;16:10926. doi: 10.15766/mep_2374-8265.10926 (PMC7412764; doi:10.15766/mep_2374-8265.10926)
Supplement: Supplementary file 1 — SP Cases.docxPE Cards.docxLogistics.docxDoor Charts.docxWorksheets.docxDebrief.docxLearner Evaluation.docx [file mep_2374-8265.10926-s001.zip › D. Door Charts.docx]

**Patient name:** Jack Simmons

**Age:** 20 years

**CC:**  abdominal pain

**Vitals:**

Temp 100.8 F

BP 135/82

HR 85

RR 14

O_2_ sat 99% on room air

**Patient name:** Amy Morton

**Age:** 45 years

**CC:**  abdominal pain

**Vitals:**

Temp 100.2 F

BP 155/80

HR 95

RR 14

Weight 160 lb. (BMI 31 kg/m^2^)

O_2_ sat 98% on room air

**Patient name:** Adam Morton

**Age:** 45 years

**CC:**  abdominal pain

**Vitals:**

Temp 100.2 F

BP 155/80

HR 95

RR 14

Weight 160 lb. (BMI 31 kg/m^2^)

O_2_ sat 98% on room air

**Patient name:** Thomas Reese

**Age:** 24 years

**CC:**  diarrhea

**Vitals:**

Temp 98.8 F

BP 125/80

HR 75

RR 14

O_2_ sat 100% on room air

**Patient name:** Tina Reese

**Age:** 24 years

**CC:**  diarrhea

**Vitals:**

Temp 98.8 F

BP 125/80

HR 75

RR 14

O_2_ sat 100% on room air

**Patient name:** Jane Anderson

**Age:** 57 years

**CC:**  poor appetite, weight loss

**Vitals:**

Temp 99.5 F

BP 145/84

HR 90

RR 14

O_2_ sat 99% on room air

**Patient name:** Joe Anderson

**Age:** 57 years

**CC:**  poor appetite, weight loss

**Vitals:**

Temp 99.5 F

BP 145/84

HR 90

RR 14

O_2_ sat 99% on room air
