# Supplementary material for: The GI Simulated Clinic: A Clinical Reasoning Exercise Supporting Medical Students' Basic and Clinical Science Integration
Source: MedEdPORTAL. 2020 Aug 5;16:10926. doi: 10.15766/mep_2374-8265.10926 (PMC7412764; doi:10.15766/mep_2374-8265.10926)
Supplement: Supplementary file 1 — SP Cases.docxPE Cards.docxLogistics.docxDoor Charts.docxWorksheets.docxDebrief.docxLearner Evaluation.docx [file mep_2374-8265.10926-s001.zip › E. Worksheets.docx]

**Jack Simmons**

| Compare and contrast findings from the diseases you’re considering to those in this patient. Note how much each disease overlaps with your patient’s case; of the overlapping features, note which ones are most distinctive to each disease in the differential. Then reorder your differential based on how well each disease matches your patient’s problem representation (with “#1” on the list being most likely/best match to your patient). | | | | |
| --- | --- | --- | --- | --- |
| **Presentation Feature** | **My Patient’s Problem Representation** | **Diseases in the Differential Diagnosis for My Patient’s Problem Representation** | | |
|  |  | **Disease A:** | **Disease B:** | **Disease C:** |
| **CC/HPI:** | **CC/HPI:** | **Presenting symptoms/HPI:** | **Presenting symptoms/HPI:** | **Presenting symptoms/HPI:** |
| Context, onset  Location  Quality  Timing (constant/ intermittent; duration; frequency)  Severity  Aggravating factors  Alleviating factors  Associated symptoms |  |  |  |  |
| **Risk Factors:** | **Other important history:** | **Risk Factors for this disease:** | **Risk Factors for this disease:** | **Risk Factors for this disease:** |
| Meds, Allergies  PMH, Surgical History  Family history  Social History |  |  |  |  |
| **Physical exam findings:** | **PE findings in this patient:** | **PE findings in this disease:** | **PE findings in this disease:** | **PE findings in this disease:** |
| Abnormal findings:  Normal/negative findings: |  |  |  |  |

| **Prioritized differential diagnosis:** List your top 3 diagnoses for this patient in order of likelihood. | **1.** | **2.** | **3.** |
| --- | --- | --- | --- |

| **Diagnostic Studies:** Select up to 5 tests that you would like to order to help make the diagnosis; note that you do not need to use all 5 testing possibilities. | **1. _____________________________**  **2. _____________________________**  **3. _____________________________** | **4. ___________________________**  **5. ___________________________** |
| --- | --- | --- |

**Amy/Adam Morgan**

| Compare and contrast findings from the diseases you’re considering to those in this patient. Note how much each disease overlaps with your patient’s case; of the overlapping features, note which ones are most distinctive to each disease in the differential. Then reorder your differential based on how well each disease matches your patient’s problem representation (with “#1” on the list being most likely/best match to your patient). | | | | |
| --- | --- | --- | --- | --- |
| **Presentation Feature** | **My Patient’s Problem Representation** | **Diseases in the Differential Diagnosis for My Patient’s Problem Representation** | | |
|  |  | **Disease A:** | **Disease B:** | **Disease C:** |
| **CC/HPI:** | **CC/HPI:** | **Presenting symptoms/HPI:** | **Presenting symptoms/HPI:** | **Presenting symptoms/HPI:** |
| Context, onset  Location  Quality  Timing (constant/ intermittent; duration; frequency)  Severity  Aggravating factors  Alleviating factors  Associated symptoms |  |  |  |  |
| **Risk Factors:** | **Other important history:** | **Risk Factors for this disease:** | **Risk Factors for this disease:** | **Risk Factors for this disease:** |
| Meds, Allergies  PMH, Surgical History  Family history  Social History |  |  |  |  |
| **Physical exam findings:** | **PE findings in this patient:** | **PE findings in this disease:** | **PE findings in this disease:** | **PE findings in this disease:** |
| Abnormal findings:  Normal/negative findings: |  |  |  |  |

| **Prioritized differential diagnosis:** List your top 3 diagnoses for this patient in order of likelihood. | **1.** | **2.** | **3.** |
| --- | --- | --- | --- |

| **Diagnostic Studies:** Select up to 5 tests that you would like to order to help make the diagnosis; note that you do not need to use all 5 testing possibilities. | **1. _____________________________**  **2. _____________________________**  **3. _____________________________** | **4. ___________________________**  **5. ___________________________** |
| --- | --- | --- |

**Thomas/Tina Reese**

| Compare and contrast findings from the diseases you’re considering to those in this patient. Note how much each disease overlaps with your patient’s case; of the overlapping features, note which ones are most distinctive to each disease in the differential. Then reorder your differential based on how well each disease matches your patient’s problem representation (with “#1” on the list being most likely/best match to your patient). | | | | |
| --- | --- | --- | --- | --- |
| **Presentation Feature** | **My Patient’s Problem Representation** | **Diseases in the Differential Diagnosis for My Patient’s Problem Representation** | | |
|  |  | **Disease A:** | **Disease B:** | **Disease C:** |
| **CC/HPI:** | **CC/HPI:** | **Presenting symptoms/HPI:** | **Presenting symptoms/HPI:** | **Presenting symptoms/HPI:** |
| Context, onset  Location  Quality  Timing (constant/ intermittent; duration; frequency)  Severity  Aggravating factors  Alleviating factors  Associated symptoms |  |  |  |  |
| **Risk Factors:** | **Other important history:** | **Risk Factors for this disease:** | **Risk Factors for this disease:** | **Risk Factors for this disease:** |
| Meds, Allergies  PMH, Surgical History  Family history  Social History |  |  |  |  |
| **Physical exam findings:** | **PE findings in this patient:** | **PE findings in this disease:** | **PE findings in this disease:** | **PE findings in this disease:** |
| Abnormal findings:  Normal/negative findings: |  |  |  |  |

| **Prioritized differential diagnosis:** List your top 3 diagnoses for this patient in order of likelihood. | **1.** | **2.** | **3.** |
| --- | --- | --- | --- |

| **Diagnostic Studies:** Select up to 5 tests that you would like to order to help make the diagnosis; note that you do not need to use all 5 testing possibilities. | **1. _____________________________**  **2. _____________________________**  **3. _____________________________** | **4. ___________________________**  **5. ___________________________** |
| --- | --- | --- |

**Jane/Joe Anderson**

| Compare and contrast findings from the diseases you’re considering to those in this patient. Note how much each disease overlaps with your patient’s case; of the overlapping features, note which ones are most distinctive to each disease in the differential. Then reorder your differential based on how well each disease matches your patient’s problem representation (with “#1” on the list being most likely/best match to your patient). | | | | |
| --- | --- | --- | --- | --- |
| **Presentation Feature** | **My Patient’s Problem Representation** | **Diseases in the Differential Diagnosis for My Patient’s Problem Representation** | | |
|  |  | **Disease A:** | **Disease B:** | **Disease C:** |
| **CC/HPI:** | **CC/HPI:** | **Presenting symptoms/HPI:** | **Presenting symptoms/HPI:** | **Presenting symptoms/HPI:** |
| Context, onset  Location  Quality  Timing (constant/ intermittent; duration; frequency)  Severity  Aggravating factors  Alleviating factors  Associated symptoms |  |  |  |  |
| **Risk Factors:** | **Other important history:** | **Risk Factors for this disease:** | **Risk Factors for this disease:** | **Risk Factors for this disease:** |
| Meds, Allergies  PMH, Surgical History  Family history  Social History |  |  |  |  |
| **Physical exam findings:** | **PE findings in this patient:** | **PE findings in this disease:** | **PE findings in this disease:** | **PE findings in this disease:** |
| Abnormal findings:  Normal/negative findings: |  |  |  |  |

| **Prioritized differential diagnosis:** List your top 3 diagnoses for this patient in order of likelihood. | **1.** | **2.** | **3.** |
| --- | --- | --- | --- |

| **Diagnostic Studies:** Select up to 5 tests that you would like to order to help make the diagnosis; note that you do not need to use all 5 testing possibilities. | **1. _____________________________**  **2. _____________________________**  **3. _____________________________** | **4. ___________________________**  **5. ___________________________** |
| --- | --- | --- |
