# Supplementary material for: The GI Simulated Clinic: A Clinical Reasoning Exercise Supporting Medical Students' Basic and Clinical Science Integration
Source: MedEdPORTAL. 2020 Aug 5;16:10926. doi: 10.15766/mep_2374-8265.10926 (PMC7412764; doi:10.15766/mep_2374-8265.10926)
Supplement: Supplementary file 1 — SP Cases.docxPE Cards.docxLogistics.docxDoor Charts.docxWorksheets.docxDebrief.docxLearner Evaluation.docx [file mep_2374-8265.10926-s001.zip › F. Debrief.docx]

**GI Simulated Clinic Debrief**

*Description and Facilitator instructions*

The debrief for the GI Simulated Clinic includes a facilitated large-group discussion of the cases presented in the SP encounter series as well as opportunities for students to ask clarifying questions about these cases.

Ideally, the facilitator should review students’ worksheets from the GI Simulated Clinic prior to conducting the debrief, if time permits. This provides the facilitator with an assessment of students’ recognition of illness scripts for the diseases represented by the SP cases, the breadth and appropriateness of their differential diagnoses, and their performance in suggesting appropriate diagnostic studies. This information can help guide the facilitator’s use of time spent on particular cases or aspects of these cases during the debrief session.

During the debrief, for each case, the large-group facilitator should start by reminding students of the case with a brief description of its major findings. Then, the facilitator should facilitate discussion of that case by prompting students with a series of questions (see suggested discussion prompts below). Each question should be used as a springboard for further large-group discussion about the differential diagnosis for each case. These discussion prompts are specifically designed to explicitly identify key and distinguishing features of each case scenario in order to ensure recognition of the illness scripts for the diseases represented by these simulated patient cases, as this was a major objective of the GI Simulated Clinic activity.

If desired, the questions below may be administered to students using commercially available audience polling software; alternatively, the facilitator may use these questions to interact with the student large group verbally. If audience-polling software is used: the facilitator can use the results of students’ responses to identify specific content areas in which further clarification is needed to rectify incomplete student understanding (i.e., to identify questions for which there is high variability and/or poor performance in student responses).

**Facilitated Discussion Prompts:**

Patient: Jack Simmons

Brief case description: 20-year-old patient with acute onset of right lower quadrant abdominal pain, fever, and nausea

1. What is the correct diagnosis?
   1. Food poisoning
   2. Acute diverticulitis
   3. Crohn’s disease
   4. **Acute appendicitis**
2. Which of the following features of the patient history was most important in making the correct diagnosis?
   1. Associated nausea
   2. Associated fever
   3. **Pain location and migration**
   4. Pain severity
3. Which of the following features of the physical examination was most important in making the correct diagnosis?
   1. Low-grade fever
   2. **Tenderness over McBurney’s point**
   3. Normal bowel sounds
   4. Lack of rebound tenderness
4. Which of the following lab tests would you expect to be abnormal in this case?
   1. **CBC (hemoglobin, hematocrit, WBC, platelet count)**
   2. CMP (electrolytes, renal function, liver function tests)
   3. Amylase, lipase
   4. Urinalysis
5. Which of the following imaging studies would be most useful in making the correct diagnosis?
   1. KUB (plain film of the abdomen)
   2. Ultrasound of the abdomen
   3. **CT of the abdomen/pelvis**
   4. MR of the abdomen/pelvis

Patient: Amy/Adam Morton

Brief case description: 45-year-old patient with acute onset, right upper quadrant abdominal pain, nausea, and vomiting

1. What is the correct diagnosis?
   1. **Acute cholecystitis**
   2. Gastric cancer
   3. Acute diverticulitis
   4. Myocardial infarction
2. Which of the following features of the patient history was most important in making the correct diagnosis?
   1. Associated vomiting
   2. Associated fever
   3. Pain location and radiation
   4. **Post-prandial timing of pain**
3. Which of the following features of the physical examination was most important in making the correct diagnosis?
   1. Low-grade fever
   2. **Positive Murphy’s sign**
   3. BMI 31
   4. Lack of jaundice
4. Which of the following lab tests would you expect to be abnormal in this case?
   1. **CBC (hemoglobin, hematocrit, WBC, platelet count)**
   2. CMP (electrolytes, renal function, liver function tests)
   3. Amylase, lipase
   4. Urinalysis
5. Which of the following imaging studies would be most useful in making the correct diagnosis?
   1. KUB (plain film of the abdomen)
   2. **Ultrasound of the abdomen**
   3. CT of the abdomen/pelvis
   4. MR of the abdomen/pelvis

Patient: Thomas/Tina Reece

Brief case description: 24-year-old patient with 2 months of progressively worsening diarrhea, now bloody, with associated abdominal cramps, and left lower quadrant tenderness

1. What is the correct diagnosis?
   1. Enterotoxigenic E. coli
   2. Ischemic colitis
   3. Crohn’s disease
   4. **Ulcerative colitis**
2. Which of the following features of the history was most important in identifying the correct cause of this patient’s bloody diarrhea?
   1. Travel history
   2. **Duration of symptoms**
   3. Fecal urgency
   4. Associated abdominal cramping
3. Which of the following features of the physical examination was most important in making the correct diagnosis?
   1. Pale conjunctivae
   2. **LLQ abdominal tenderness**
   3. Lack of rebound tenderness
   4. Rectal examination
4. Which of the following lab tests would be most important in making the correct diagnosis?
   1. CRP (C-reactive protein)
   2. Rectal swab for Chlamydia trachomatis/Neisseria gonorrhea DNA
   3. **Gastrointestinal Pathogen Panel**
   4. FIT test (fecal immunochemical test for occult blood)
5. Which of the following imaging studies would be most useful in making the correct diagnosis?
   1. CT colonography (Virtual Colonoscopy)
   2. **Colonoscopy**
   3. CT of the abdomen/pelvis
   4. MR of the abdomen/pelvis

Patient: Jane/Joe Anderson

Brief case description: 57-year-old patient with 3 months of poor appetite, a 20-pound unintentional weight loss, pruritus, scleral icterus, and jaundice.

1. What is the correct diagnosis?
   1. Gastric cancer
   2. **Pancreatic cancer**
   3. Choledocholithiasis
   4. Acute hepatitis
2. Which of the following features of the patient history was most important in making the correct diagnosis?
   1. **Associated weight loss**
   2. **Associated jaundice**
   3. Absence of abdominal pain
   4. Anorexia
3. Which of the following features of the physical examination was most important in making the correct diagnosis?
   1. Skin excoriations
   2. Normal bowel sounds
   3. Lack of abdominal tenderness
   4. **Jaundice**
4. Which of the following lab tests would you expect to be abnormal in this case?
   1. CBC (hemoglobin, hematocrit, WBC, platelet count)
   2. **CMP (electrolytes, renal function, liver function tests)**
   3. Amylase, lipase
   4. Pregnancy test
5. Which of the following imaging studies would be most useful in making the correct diagnosis?
   1. KUB (plain film of the abdomen)
   2. **Ultrasound of the abdomen**
   3. **CT of the abdomen/pelvis**
   4. MR of the abdomen/pelvis
