# Supplementary material for: The GI Simulated Clinic: A Clinical Reasoning Exercise Supporting Medical Students' Basic and Clinical Science Integration
Source: MedEdPORTAL. 2020 Aug 5;16:10926. doi: 10.15766/mep_2374-8265.10926 (PMC7412764; doi:10.15766/mep_2374-8265.10926)
Supplement: Supplementary file 1 — SP Cases.docxPE Cards.docxLogistics.docxDoor Charts.docxWorksheets.docxDebrief.docxLearner Evaluation.docx [file mep_2374-8265.10926-s001.zip › G. Learner Evaluation.docx]

**Learner Evaluation**

The following survey questions refer to the recent simulated GI clinic in which you participated.  Your responses on this survey will be used to evaluate how we provide this instruction for future medical students.  Your instructors will not know whether you completed this survey or how you answered the questions.  Your identity will remain confidential, and your responses will have no impact on your course grade.

How relevant was the content of this learning activity to your role as a future physician?

- Extremely relevant
- Quite relevant
- Moderately relevant
- Slightly relevant
- Not at all relevant

Was the small-group format of this learning activity appropriate for the learning content presented?

- Yes
- No

Were the pace and duration of this learning activity appropriate for the learning content presented?

- Yes
- No

Was the problem-solving aspect of this learning activity intellectually stimulating?

- Yes
- No

Do you prefer classes that include this type of small group problem-solving activity rather than classes that do not include this type of activity?

- Yes
- No

**Comments:**

**How effective were each of the following aspects of the GI simulated clinic activity?**

Effectiveness of standardized patient (SP) encounters for learning about the clinical features of the diseases presented in this learning activity:

- Extremely effective
- Quite effective
- Moderately effective
- Slightly effective
- Not effective at all

Effectiveness of the SP encounters for practicing your diagnostic reasoning skills related to the diseases presented in this learning activity:

- Extremely effective
- Quite effective
- Moderately effective
- Slightly effective
- Not effective at all

Effectiveness of the SP interviews for reinforcing history-taking skills you have learned in the Clinical Skills course:

- Extremely effective
- Quite effective
- Moderately effective
- Slightly effective
- Not effective at all

Effectiveness of the SP encounters for reinforcing physical examination skills you have learned in the Clinical Skills course:

- Extremely effective
- Quite effective
- Moderately effective
- Slightly effective
- Not effective at all

**Comments:**

**What was the overall effectiveness of the GI simulated clinic activity** (1 = poor; 10 = excellent)?

- 10
- 9
- 8
- 7
- 6
- 5
- 4
- 3
- 2
- 1

**Which aspects of this learning event were most effective?**

**Which aspects of this learning event could be improved?** (Please provide specific suggestions):

**Would you like to participate in additional SP encounter learning activities, structured like this one?**

- Yes
- No
